# The role of network bridging organisations in compensation payments for agri-environmental services under the EU Common Agricultural Policy

**DOI:** 10.1016/j.ecolecon.2015.07.025

**Published:** 2015-11

**Authors:** Tom Dedeurwaerdere, Audrey Polard, Paolo Melindi-Ghidi

**Affiliations:** a Université catholique de Louvain, Belgium; b National Research Foundation, F.R.S.-FNRS, Belgium; c Aix-Marseille Université, GREQAM, France

**Keywords:** Payments for environmental services, EU Common Agricultural Policy, Bridging organisations, Network governance, Social learning, Environmental attitudes

## Abstract

Compensation payments to farmers for the provision of agri-environmental services are a well-established policy scheme under the EU Common Agricultural Policy. However, in spite of the success in most EU countries in the uptake of the programme by farmers, the impact of the scheme on the long term commitment of farmers to change their practices remains poorly documented. To explore this issue, this paper presents the results of structured field interviews and a quantitative survey in the Walloon Region of Belgium. The main finding of this study is that farmers who have periodic contacts with network bridging organisations that foster cooperation and social learning in the agri-environmental landscapes show a higher commitment to change. This effect is observed both for farmers with high and low concern for biodiversity depletion. Support for network bridging organisations is foreseen under the EU Leader programme and the EU regulation 1306/2013, which could open-up interesting opportunities for enhancing the effectiveness of the current payment scheme for agri-environmental services.

## 1 Introduction

Research over the last two decades has shown that human influences on global life-support systems have reached a magnitude unprecedented in human history (Jerneck et al., 2010). On the one hand, pro-growth economic policies have encouraged technological innovations and rapid accumulation of consumption goods (Komiyama and Takeuchi, 2006, Orecchini et al., 2012). This resulted in increased human prosperity in many parts of the world, although in a globally disproportionate manner. On the other hand, by depleting the world's stock of natural wealth on a global scale – often irreversibly – the prevailing, and predominant, economic and development models have increasingly detrimental impacts on the wellbeing of present generations, in particular leading to a broadening ecological crisis and ever-widening social disparities. Concomitantly, these models present tremendous risks and challenges for future generations.

In response to these rapid changes, policy makers, in conjunction with researchers and civil society organisations, have organised over the last three decades vast scientific assessment efforts (Haas, 2004), developed a growing body of environmental law-making and have set up environmental bureaucracies to implement new regulatory regimes. However, in spite of important progress in many areas, the situation of rapidly degrading ecosystem services has not been reversed. The situation is worrisome, in particular because most of the driving forces of environmental change, such as economic growth, resource use and energy consumption, continue to increase (Jaeger, 2011).

Two major reasons for the lack of significant progress highlighted by sustainability scholars are, first, the poor integration of environmental policies with other policy fields and, second, the failure of conventional expert-led and state-centred governance regimes to deal with highly uncertain and complex transition processes. First, the lack of integration of environmental policies with other fields leads to lock-in in unsustainable socio-ecological states, as progress in environmental sustainability can be hampered by the interdependence between natural resource regimes, technological infrastructure and socio-economic patterns of consumption and production (Arthur, 1994, Smith et al., 2005, Geels and Schot, 2007). What is needed to overcome such lock-in are governance regimes which are not only functionally stable in each of their sub-systems, but which generate a societal transition in a convergent and mutually supporting way between the various sub-systems (Pahl-Wostl, 2007a, Pahl-Wostl, 2007b). Second, many of the sustainability problems are characterised by scientific uncertainty and complexity. In particular, knowledge about the dynamics of socio-ecological systems is dispersed amongst local, regional and national agencies and groups. For such problems, state-centred and expert-led approaches to transition alone – in spite of their important role in building convergent and evidence-based knowledge (Haas, 2004) – have been shown to be insufficient to generate the necessary societal change (Berkes, 2009). In response to these challenges, both social actors and policy makers have called for increasing cooperation and social learning amongst state and non-state collective actors.

This paper aims to contribute to the growing literature on the role of collaborative networks of state and non-state collective actors in policy integration and social learning for policy integration in the field of sustainability transitions. Such collaborative networks have emerged in the 1990s as an important complement to the conventional market-based or regulatory forms of governance. These collaborative networks might include collaborative forms of governance amongst and within state organisations, environmental and socio-cultural associations, research institutions, worker unions, employers' federations and social cooperatives amongst others (Kanie and Haas, 2004, Delmas and Young, 2009). As shown in the literature, the combination of markets, governmental hierarchies and networks is especially important to improve the effectiveness of environmental policies (Dedeurwaerdere, 2005a, Dedeurwaerdere, 2005b).

The study focuses in particular on one prominent case of the integration of environmental policies with other policy fields through such collaborative networks, namely, agri-environmental measures in the context of the EU Common Agricultural Policy. Environmental policy integration is part of the constitutional requirements of the EU (as specified in the Treaty on the functioning of the EU) and has to be applied in particular to the Common Agricultural Policy (Mestre et al., 2011). In practice, agri-environmental policy contributes to policy integration through a policy of payments to farmers for voluntary measures to implement environmental farming practices. As argued in the literature on payments for environmental services, such voluntary payment schemes are expected to contribute to environmental goals at low costs and without introducing additional direct regulations, to enlist state, market, non-profit organisations and civic actors in the design and delivery of public policy, and to support economic growth, while still achieving regulatory and conservation goals (Paavola and Hubacek, 2013).

In spite of this well-established scheme within the Common Agricultural Policy, the implementation of agri-environmental measures in general mainly proceeds according to a traditional state-led and expert-led mode of governance and fails to achieve the stated goals of integration. Indeed, implementation is often managed by a centralised follow-up committee appointed in each member state (or the regional authority in the member state), which is characterised by a top-down approach of design and monitoring of the scheme (Morris, 2006). However, the top-down approach does not address the social learning needs amongst the multiple stakeholders operating in the agri-environmental landscapes. As a result, in spite of the relative success in the uptake of this scheme throughout Europe, farmers who adopt agri-environmental measures tend to conform to the requirements of the scheme only formally, but do not necessarily embark upon a social learning process that contributes to an integration of the environmental practices with other practices in the agro-environmental landscape and to a long-term change in agricultural practices.

Based on this literature, the hypothesis of the paper is that a policy of economic compensation payments alone for the provision of environmental services will not be enough to overcome the insufficiencies of the direct regulation approach to environmental public goods provision. To reach the goals of more sustainable agri-environmental management, the important issues of multi-stakeholder cooperation and social learning in collaborative networks of state and non-state collective actors (hereunder designated by “collaborative networks”) also need to be addressed. To test this hypothesis and to evaluate the possible role of these collaborative networks in addressing the challenges of agri-environmental service provision, this paper analyses a series of in-depth field interviews and a quantitative survey with farmers in the Walloon Region of Belgium who participate in the agri-environmental payment scheme. The paper is organised as follows: The second section discusses the possible contribution of the collaborative networks in improving the environmental effectiveness of the agri-environmental payment scheme. The third section presents a specific set of collaborative network organisations, which are the network bridging organisations, and explains how such bridging organisations can help to address the important challenge of knowledge co-production and exchange in these collaborative networks, both in an economically efficient and socially legitimate way. The fourth section presents the materials and methods of the empirical fieldwork and the survey. Section five presents and discusses the main results. The policy recommendations that result from the analysis are discussed in the electronic supplementary material provided with the article.

## 2 The Role of Collaborative Networks in Building Partnerships for Environmental Public Goods Provision in Agriculture

In the countries of Western Europe, mechanisation of agriculture and the massive use of chemical inputs have led, at least since the Second World War, to the intensification of agricultural production systems, higher levels of specialization and an increase in size of farms and farm plots. This in turn has led both to a dramatic increase in agricultural output and to serious negative consequences for the environment. The Common Agricultural Policy, put into place at the beginning of the 1960s, is a major driver of this process (Posthumus and Morris, 2010). One prominent and well-documented illustration is the detrimental impact on farmland bird populations (Butler et al., 2010). Between 1980 and 2009, the farmland bird population has decreased in Europe from 600 million to 300 million, implying a loss of 50%. The removal of hedgerows and the ploughing over of meadows are two significant factors that have contributed to more efficient farming, but they have also contributed to the decrease in farmland birds' habitats.

To take into account this and other detrimental environmental impacts of agriculture policies, environmental organisations and policy makers have advocated a series of reforms of the EU Common Agricultural Policy. In particular, the 1992 MacSharry reforms, which introduced agri-environmental schemes (AES), played a major role in the efforts to alleviate these detrimental impacts. However, as has also been discussed elsewhere, other factors also played a role in the adoption of these reforms (Burton and Schwarz, 2013). In particular, both the decision to have recourse to compensation payments as the main tool for the EU agri-environmental policy and the design of these payment schemes were influenced by the negotiations of the Uruguay round and the subsequent rules adopted under the WTO agreement. In particular, WTO requirements lead to use action-oriented payments, based on compensation payments for the delivery of specific land management practices, instead of outcome-based measures focused on the provision of environmental outcomes. In addition, under the WTO rules, any compensation for the services delivered should remain limited to the additional cost of compliance incurred. In spite of some obvious limits of the scheme (Berendse et al., 2004, Goetz and Brouwer, 2010), such as its limited action on the realisation of long-term attitudinal changes of farmers, the action-based approaches have become the dominant means of securing environmental public goods in Europe.

With no major alterations to the WTO agreement, this approach is likely to remain a key policy environmental tool and there is a well-recognised need for further improvements of the design, targeting and implementation of the agri-environmental measures (Zabel and Roe, 2009, Herzon and Mikk, 2007). Two important implementation challenges, which hamper the further improvement in the effective realisation of the stated environmental objectives, are the focus of this paper and will be analysed in relation to the agri-environmental measures which have been proposed in the Walloon Region of Belgium in the 5-year period with the farmers running from 2007 to 2013 (cf. Table 1).

The first challenge is related to the nature of the environmental requirements of the measures (defined as the gap between the specifications of the measures and the general legal compulsory baseline related to good agri-environmental practices). Some measures under the scheme impose a higher level of environmental requirements to the farmers (which we will call hereunder “medium” or “deep green” agri-environmental measures), while others impose a lower level of environmental requirements (which we will call “light green” agri-environmental measures), as illustrated in Table 1. Even if a higher environmental outcome will already be reached when more farmers adopt more measures, a better result will be obtained if the proportion of medium/deep measures can be increased as the latter lead to higher environmental outcomes. However, in practice, a lot of farmers tend to adhere only to the light green measures and fewer farmers adhere in addition to the medium or deep green (Defrancesco et al., 2008). This might be related to the difficulty to apply the medium/deep measures on certain farms (as some of these measures require a pre-existing ecological potential) or to the willingness of the farmer to accept the environmental service provision contract in spite of a given compensation payment (which is adjusted, as best as possible, to the average economic opportunity costs for the farmer).

The second challenge is related to the long-term change in agricultural practices. A substantial change in practices by the farmers is one of the important means envisioned in the scheme for better reaching the environmental objectives (Zabel and Roe, 2009). Indeed some measures can be implemented through simply maintaining the existing practices on the farm (for instance, continuing to maintain existing hedgerows). This might already be considered as a positive result of the payment scheme. But in many situations a change towards more environmental practices is required to effectively implement a measure or to improve the environmental outcomes in the agri-environmental landscape. For instance, for some measures, the environmental impact may depend on the action on several farms and not just one. In other cases, the farmer might chose only to keep existing hedgerows when applying to the scheme or to plant new hedgerows on his farm, when this allows to better connect parcels of ecological interest in the landscape. However, adhesion to the scheme is not always linked with such a change in environmental practices by farmers, even for those farmers who adhere to the deep or medium measures, where such change is feasible on the farm or in cases where it is an essential component of effective implementation (Burton et al., 2008, Poláková et al., 2011).

To overcome these shortcomings, scholars have highlighted the need to look beyond the formal aspects of the governmental payment scheme. Indeed, actors that participate in payment schemes for environmental services are in part motivated by the monetary compensation, but may also be motivated by the satisfaction of social norms and personal values (Morris and Potter, 1995, Defrancesco et al., 2008, Burton and Paragahawewa, 2011, Rico García-Amado et al., 2013, Mills et al., 2013, Primmer et al., 2014). This is in particular true in the case of agri-environmental public goods, where social–cultural and ecological value dimensions play an important role, in addition to the economic value dimensions (cf. De Groot and Rudolf, 2002, De Groot and Rudolf, 2006). When social norms and personal values play an important role in the decision-making over alternatives (under similar economic outcomes, and similar level of information), collaborative networks that build trust amongst the actors and foster learning of the new social norms (Muradian and Rival, 2012) have been shown to be more likely to lead to change in actors' behaviour (Dedeurwaerdere, 2005a, Dedeurwaerdere, 2005b, Rydin, 2006, Dedeurwaerdere, 2007, Innes and Booher, 2010, Bodin and Prell, 2011).

In the field of study of environmental governance, collaborative networks can be defined through three distinguishing features (Pahl-Wostl, 2009):

1) a high level of social regulation through informal institutions, which depend on social norms for rule enforcement, in addition to formal institutions, which are controlled and monitored by the legal apparatus of the state;
2) a high degree of participation of a broad set of stakeholders in the governance process and
3) the recourse to interactive problem solving amongst a decentralised network of collective actors.

Empirical evidence supports the contribution of these features to improving environmental outcomes. First, governance networks have proven to be essential in situations of heterogeneity amongst the concerned actors (Nooteboom, 2006, Olsson et al., 2006). In particular, they may be very flexible in terms of enrolment of new participants, and in terms of the role and type of actors and connections. Moreover, because of the absence of formal control, they may be much more open-ended, in particular by supporting multiple ways of envisioning and operationalising various transition pathways. Second, broad participation in the environmental policy schemes can reduce the likelihood of unexpected resistance in the implementation (Berkes et al., 2003). As has been shown in the vast literature on community produced public goods, participation can lead to increased compliance and effectiveness with the common rules for sustainable use of common pool resources (Ostrom, 1990). Third, including a broader set of stakeholders gives access to different kinds of knowledge which may be vital for finding innovative solutions (Berkes and Folke, 2002). Finally, modular system structures and decentralised control can lead to higher degree of adaptiveness and robustness of a system (Pahl-Wostl, 2009; Miller and Page, 2007). However, governance networks based on these features can also fail, suffer from problems of legitimacy or from high implementation costs. The key message from the literature is therefore that a more diversified governance system, which has recourse to government, market and collaborative networks, will lead to a higher adaptive capacity for tackling complex socio-ecological problems.

In order to explore the impact of the participation of farmers in such collaborative networks on their adhesion to the agri-environmental scheme and their environmental efforts, a better understanding of the roles of these networks is needed. In particular, as highlighted above, collaborative networks are needed that address the social learning needs amongst the various actors in the agri-environmental landscapes and a more effective cooperation amongst conventional expert-led knowledge production on the one hand and the practitioners' knowledge on the other. This paper will therefore focus on one particular type of collaborative network organisation, which is the network bridging organisation (also called boundary organisation). The specific role of this type of organisation is to facilitate the co-production and exchange of knowledge amongst actors within the network with different cognitive background frames. The next section addresses the role of these network bridging organisations from a theoretical perspective, with the view to understanding their contribution to knowledge co-production and exchange on environmental public goods both from the point of view of the efficiency (theory of public good economics) and social legitimacy (theory of social learning).

## 3 The Contribution of Network Bridging Organisations to Environmental Public Goods Provision

The provision of environmental public goods through private actors, such as the environmental services in the agricultural landscapes, involves complex forms of collaborative governance with individual providers, users, stakeholders and government. In this context, many studies have focused on one particular form of collaborative governance, which has proven particularly effective. This is formal co-management with a high level of officially agreed power-sharing amongst the government and the user/stakeholder groups (Berkes, 2009, Dedeurwaerdere, 2009). However, formal co-management regimes between the state and user/stakeholder groups are not well developed within the context of the implementation of the Common Agricultural Policy.

The networks of state and non-state collective actors that emerged in response to the participatory governance challenges of the agri-environmental scheme are situated somewhere in between the formal co-management and a purely advisory model of co-governance. An example of an advisory model of co-governance is the environmental forum in Germany, which has been put in place in the context of the agri-environmental policy (Prager and Nagel, 2008). In such an advisory model, the non-state actors are consulted by a regulatory/centralised state, but have no decision-making power or direct impact on the implementation decisions beyond the consultative mechanism. In contrast, the collaborative networks establish a horizontal form of cooperation amongst a diversity of actors and the state, which directly influences the implementation of the policies for the provision of environmental public goods. The respective roles of these different forms of co-governance in agri-environmental public goods provision are illustrated in Table 2.

**Fig. 1:**
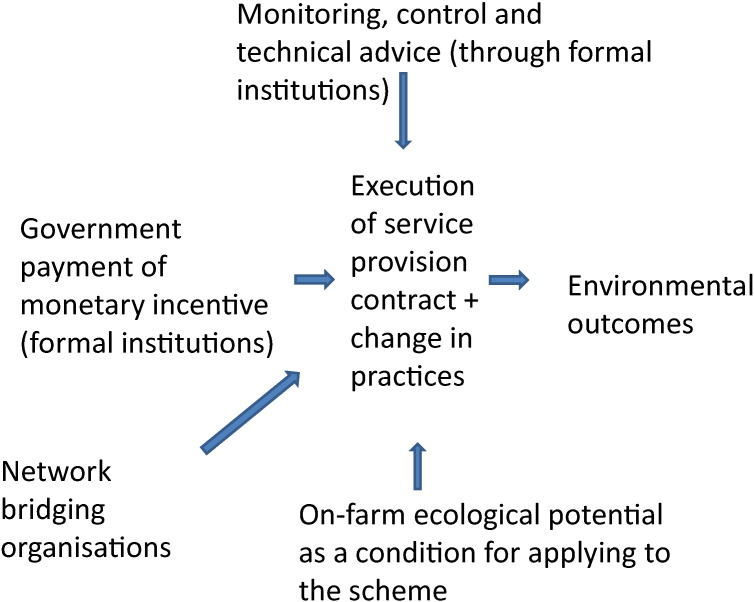
The role of bridging organisations in social learning and knowledge co-production on agri-environmental public goods (PG).

Adapted from Berkes (2009, p. 1696).

However, collaborative networks on their own are unlikely to overcome the challenges raised by environmental public goods provision. Indeed, as highlighted for instance in the review paper by Berkes (2009), managing the provision of environmental public goods through collaborative governance is an information-intensive endeavour. In many contexts, the different actors need to work and think together based on knowledge from different sources (from scientists, practitioners and governmental expertise), deliberate to generate new knowledge and engage in social learning over new normative orientations and common understandings. This complex process of knowledge provision in situations of collaborative governance therefore often relies on so-called bridging organisations, which organise knowledge co-production and social learning amongst the various actors and types of knowledge. Fig. 1 illustrates the role of such bridging organisations for the specific case of agri-environmental public goods provided by farmers. As can be seen from this figure, the possible types of knowledge co-produced by such bridging organisations can be very broad and no single organisation is likely to supply all of these functions. Therefore, in practice, a number of organisations, which might also pursue other objectives in the same time, can fulfil the various roles of a bridging organisation.

The role of bridging organisations has been extensively studied in the literature in environmental governance in the specific context of formal co-management arrangements between the state and local communities or user groups (cf. Berkes, 2009, Dedeurwaerdere, 2009). Less attention has been given to the possible role of bridging organisations in the case of collaborative actor networks. Two of the merits of bridging organisations amongst participants of collaborative networks (which we designate by the term network bridging organisations) seem especially relevant for improving the effective implementation of agri-environmental policies and will be discussed in the remainder of this section. The first is the improvement in the level of environmental public goods provision by exploiting the complementarities in the available expertise between governmental agencies and various non-state actors that contribute to the public good provision. The second is the potential for facilitating the process of social learning and co-production of knowledge amongst the stakeholders, private sector actors and local/regional or national governmental organisations.

### 3.1 Exploiting Knowledge Complementarities in the Collaborative Networks

Scholars in public good economics have elaborated a set of basic models to explain the existence and the efficiency benefits of cooperation in collaborative actor networks (Weisbrod, 1988). The basic economic approach to the complementary between the various state and non-state actors contributing to the provision of public goods is based on the concept of demand heterogeneity for public goods. Demand heterogeneity is defined by the fact that different population groups (such as pro-environmental groups versus rural development organisations) have divergent demands for public goods in both quantity and quality. They can for instance agree upon a basic level of pollution mitigation, but might disagree on the priority of other aspects such as animal welfare, improvement in the quality of agricultural products or preservation of cultural heritage. Under the hypothesis where the government first attempts to satisfy the demands of the statistically average person, this can lead to a set of unmet demands of these social groups. These unmet demands might be satisfied by highly motivated individuals, voluntary initiatives of firms or by non-profit organisations, which are established and financed by the voluntary contributions (in terms of money or participation to activities) of citizens and stakeholders who want to increase the output or the quality of the public good (Anheier, 2005).

Based on these premises of public good economics, one would expect national/regional governments to focus their implementation efforts of agri-environmental measures on a set of measures that reach the largest number of farmers, which requires measures that do not go too far above the existing baseline of the general compulsory legal baseline of good environmental practices. As highlighted above, the national/regional governments' implementation of the EU agri-environmental payment scheme has indeed led to a higher investment in the light measures, in spite of the obligation under EU rules to address at least some of the deep and medium measures as well.

However, in parallel to this first trend, the unmet social demands of a more full-fledged implementation of agri-environmental policy, advocated by highly environmentally motivated citizens and social actors, have led to the emergence of a thriving non-profit sector that provides technical services, knowledge sharing and advice for improving the provision of the various agri-environmental public goods. The existence of these non-profit service-providing organisations in turn opens up interesting possibilities for cooperation between governmental organisations, non-profit organisations and research institutions (Salamon, 2002, Anheier, 2005, ch. 13).

On the one hand, the non-profit organisations and research institutions at universities have developed a set of capacities that can provide resources to the government for improving its efficiency in implementing its own public good policies. Well documented examples are the capacity of these organisations to cooperate with various actors in the agri-environmental landscape level and their in-depth knowledge of the local contexts in which the farmers operate (Prager and Nagel, 2008). In addition, the non-profit organisations and autonomous research entities are in a better position to inspire trust and to play a facilitating role in social learning with the farmers, compared to private sector operators that would be paid by the state to provide an equivalent level of knowledge and technical services to the farmers (Anheier, 2005, ch. 13). On the other hand, however, non-profit organisations and research entities might be subject to a set of governance failures, such as lack of broader social accountability or lack of financial sustainability. The latter failures might be alleviated in the partnership with the state organisations (Ibid.).

The mobilisation of non-profit organisations is not without risk, however. By involving non-profit organisations in knowledge co-production and exchange, governmental actors might fear losing some public control over the process and face increased monitoring costs (Kramer, 1987). Moreover, non-profit organisations “may be forced to conform to standards imposed by contracting policy at the expense of their own notions of what constitutes efficient delivery” (Anheier, 2005, p. 289). In other words, if the funding of the non-profit organisation becomes entirely dependent on the state, there is a risk that the non-profit organisation strays from its intended mission. Therefore, an appropriate balance will need to be maintained in the non-profit organisation between the three main funding streams of membership contributions (in fees and time), donations/market services (in money and in kind) and governmental subsidies/support.

The economic model of the complementarity amongst the various types of actors operating in the collaborative networks therefore provides a first set of arguments that allow an understanding of the possible role of network bridging organisations: they can contribute to more systematically exploiting the knowledge complementarities between the state, non-profit actors and research institutions, and play the role of a trusted intermediary which provides for the necessary guarantees for preserving the autonomy of these actors.

### 3.2 The Role of Network Bridging Organisations in Social Learning

The public economic perspective, albeit useful to explain the possible role of the network bridging organisations in more efficient knowledge co-production and exchange, falls short of addressing the deeper problem of scientific uncertainty and social controversy over the best available socio-ecological transition pathways. In particular, the economic efficiency perspective presupposes that the meaning, the normative goals and the level of the public good provision are well known and well defined, as in the expert-led and top-down approaches to agri-environmental policy. Nevertheless, policymakers, researchers, farmers, private sector organisations and stakeholders are all confronted with persistent uncertainties and social controversies pertaining to agri-environmental public goods, whose effects are often of a complex multi-scale and multi-faceted (social, environmental, economic) nature. Therefore, the increase in efficiency through collaborative networking is likely to succeed only if appropriate social learning processes are put in place that lead to convergent cognitive background understandings (both factual and normative) across the various actors and scales.

The social learning challenge seems especially hard to tackle in the case of agri-environmental measures under the Common Agricultural Policy (CAP). Indeed, as seen above, many payment schemes only require farmers to formally implement the required measures, but do not address more in depth transformation of the agricultural practices on a landscape scale or do not support collaborative learning on the relevant agri-environmental issues in a given landscape. In contrast, the experiments with landscape-level implementation of agri-environmental measures highlighted above, which are based on multi-stakeholder collaboration mechanisms and social learning, have shown a higher involvement by the participating farmers in supporting long term change.

For instance, in Ontario, Canada, a system of payment for environmental services was jointly administered by the government, a coalition of 30 farm organisations throughout Ontario and an association for soil and crop improvement with farmer experts (Grudens-Schuck, 2000). In particular the local branches of the Soil and Crop Improvement Association provided for social learning through supporting the farmers with the writing of environmental action plans, providing advice through workshops and follow-up of the implementation. In this successful scheme, which at present reaches over 20,000 farms, the government plays a role which is purposefully not in a pronounced controlling function, but limited to an advisory and funding role. Other cases documented in the literature are the role of the sheep owner and cultural association in the Altmühltal in Southern Germany (OECD, 2013, p. 198) — which coordinated the application for agri-environmental payments on a regional scale, the collective approach to the implementation of agri-environmental measures in the Netherlands through regional groups and cooperatives (OECD, 2013), and the successful collaboration between reindeer herders and the authorities in Finland to restore nesting of the golden eagle through a participatory scheme, after years of conflict-ridden implementation of the compensation payment policy (Suvantola, 2013).

Social learning in collaborative networks is however not an automatic result of the existence of these networks, nor is it necessarily part of the mission of the various actors that compose the network. The literature on social learning in the management of natural resources has highlighted a set of conditions for social learning processes to occur. In particular, in their review of over two decades of empirical research with collaborative processes, Innes and Booher (2010) highlight three key conditions for successful social learning processes. These conditions are: inclusiveness, interdependence and authentic dialogue. First, to lead to effective social learning on the landscape level, a collaborative rational process has to engage all those who have pertinent knowledge and a stake in the issue at hand. Indeed, the long-term adaptive capacity of complex socio-ecological systems is fostered by a dialogue amongst a broad diversity of values, interests, perspectives, skills as well as sources of knowledge. As also highlighted by other scholars of social learning, diversity allows to uncover contradictions and differences, which in turn foster joint learning processes that take into account such social divergences. Second, to maintain the interest and energy to engage in the process, in spite of the evident costs, the participating actors need to expect higher outcomes from interdependent action within the collaborative process than from staying outside or relying on conventional direct governmental regulation or interest-based lobbying. Third, the social learning process needs to be considered legitimate by all the participating actors, through a mechanism that is not based on the conventional legitimacy provided by elected governmental bodies, but on the quality of the deliberative processes amongst the actors. According to Innes and Booher, such “deliberative” legitimacy will be provided if the interested parties are able to deliberate together in a “non-coercive environment” with valid information and with a view to reaching agreement on actions to undertake (p. 204).

**Fig. 2:**
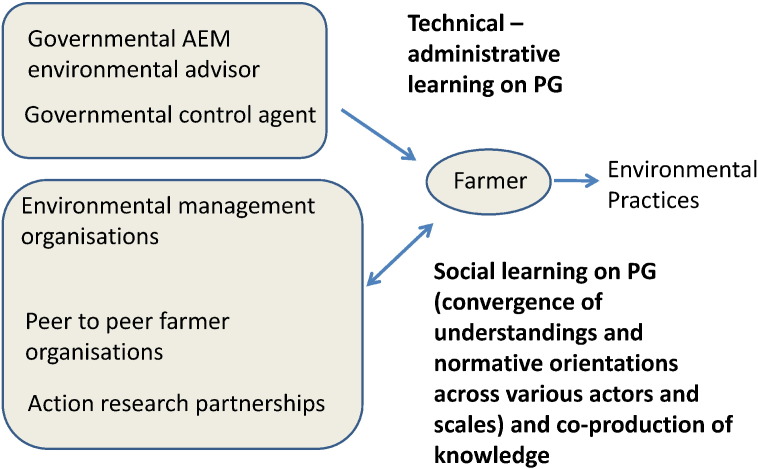
The role of network bridging organisations in social learning on agri-environmental public goods provision (PG).

These three core conditions of effective social learning have been observed in many well-documented cases of successful collaboration in natural resource management. In the context of the Common Agricultural Policy, collaborations amongst multiple stakeholders in environmental management organisations, such as in the German Altmühltal mentioned above (OECD, 2013), are good examples where the interaction amongst a high diversity of interdependent actors around agri-environmental issues, in a non-coercive environment, has led to a robust social learning process. In contrast, governmental advisors employed under the agri-environmental scheme have a much more ambiguous role, as they can be associated with the control functions of the government, which might hamper their role as a facilitator of “non-coercive” and open-ended social learning processes. This is probably one of the reasons why, in many countries, the organisation of such advice has been outsourced to non-profit organisations focused on nature protection or to universities. In addition, many other non-profit organisations contribute to knowledge co-production amongst the multiple stakeholders, even though they often lack the resources for doing so. For instance, in Canada, a highly successful governmental policy for the implementation of environmental practices in farming practices is based on support to learning processes in clubs of farmers that exchange technical knowledge on a peer to peer basis (Mathe and Rivaud, 2009). Such clubs exist in many EU countries as well (cf. the CETA and COMICE, mentioned in Table 3 of the questionnaire below). However, in many countries these peer to peer farmer organisations are not focused on environmental issues, and, if they are, lack political and financial support (Mathe and Rivaud, 2009). The various possible efficiency enhancing (technical/administrative) and social learning roles of collaborative network organisations in agri-environmental landscapes have been represented schematically in fig. 2.

## 4 Materials and Methods

To assess the potential contribution of the network bridging organisations in the effective implementation of the agri-environmental policy, this paper focuses in particular on one case, which is the agri-environmental scheme of the Walloon Region (Belgium) in the 5-year period with the farmers from 2007 to 2013 (Walloon Rural Development Programme: Gouvernement Wallon, 2008). For comparative purposes this study included all the measures of the programme, with the exception of the AEM11 which applies specifically to conversion to and support for organic farming.

In particular, through analysing a series of structured in-depth field interviews with farmers, this paper aims to better understand the incompleteness of the direct governmental regulation approach and the possible contribution of the network bridging organisations to the provision of agri-environmental public goods. For this purpose, this study uses a model which combines explanatory variables from both these approaches: variables related to the governmental compensation payment scheme (the monitoring scheme, the monetary compensation and the on-farm conditions that determine if farmers are “eligible” to the scheme) and variables to identify the role of various network bridging organisations (Fig. 4). In this model these variables jointly contribute to two outcome variables:

(a) a first outcome variable related to the decision of farmers to enter into the environmental service provision contract and
(b) a second outcome variable related to the change in the farmers' practices in direct relation to the implementation of these contracts.

**Fig. 3:**
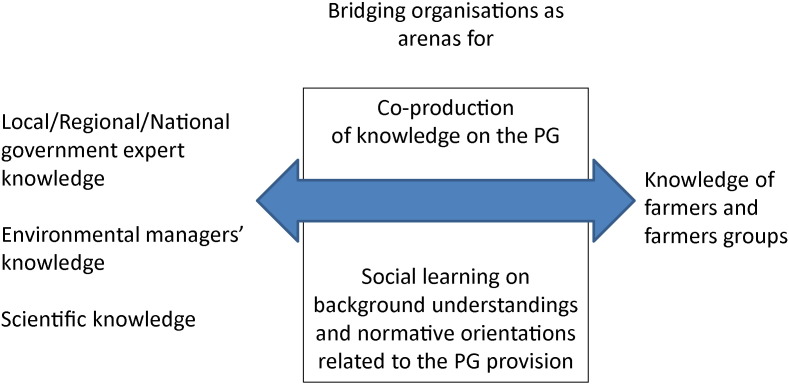
Combining direct regulation and network governance in agri-environmental policy.

**Fig. 4:**
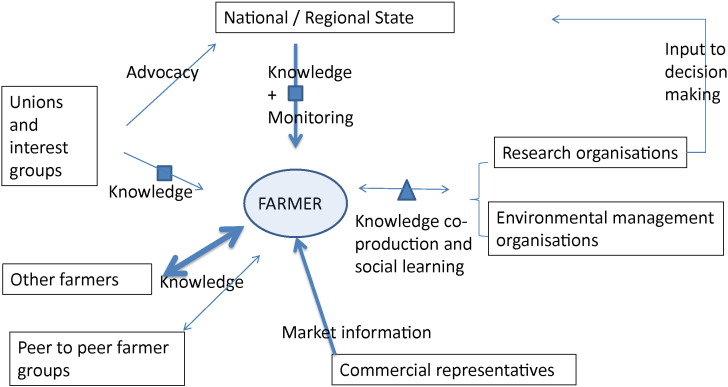
Knowledge network around the farmers involved in AEM in the Walloon Survey (data from the figures calculated manually from the data from questions 62 and 65 of the survey, cf. Annex 2; national/regional state includes the governmental agri-environmental advisors). Legend: Thickness of the arrows represents the frequency of the contacts: thickest arrow > 90% respondents indicated to have a contact at least 1/year; intermediary thickness (between 70 and 90%), thin arrow (between 50 and 70%). Triangle/square: respondents indicated that a certain percentage of the interaction concerned the follow-up of environmental practices (over 40% of the interactions included an environmental knowledge interaction (triangle), between 20% and 40% (square), the others less than 20%).

The environmental service contracts and the change in farmers' practices in direct relation to these contracts are supposed to contribute to a set of environmental outcomes. The latter are not directly part of this study, as the relation between adhesion and production of environmental outcomes has been analysed extensively elsewhere (Berendse et al., 2004) and is the object of regular compulsory evaluations and adjustments of the scheme by the EU member states, including for the Walloon agri-environmental scheme. Fig. 3 schematically represents these variables of the model.

The structured interviews explored farmers' choices for executing environmental service provision contracts in a situation where these farmers can adhere to one or several of the 13 agri-environmental measures defined in Table 1 above. The information on the adhesion of each farmer to the measures was double-checked with information provided by the Walloon Region and that was available to the authors on a confidential basis.

Structured questionnaires were used in this study to analyse the farmer decisions on each of these 13 proposed measures. To understand the impact of the governmental compensation scheme, variables were included to account for the following factors: (1) likeliness to be eligible to apply for the measures of the scheme (defined by the government in function of the ecological potential for each measure), (2) transaction costs, (3) the level of the compensation payment and (4) the role of the governmental advisers (inter alia based on the studies reported in Bonnieux and Dupraz, 2007). Control variables were included in the study to check for specificities related to the type of production system and for the farmers' motivations (to take into account the results from the literature on biodiversity contracting reported in Primmer et al., 2014). Prior to administering the questionnaire, a set of 13 in depth interviews were conducted with the agri-environmental advisors of the programme and key selected experts. The study was organised in a qualitative and a quantitative part.

The qualitative part consisted of in-depth, “on-the-farm”, interviews with 34 farmers using a common structured questionnaire, from April to July 2013. The 34 qualitative “on-the-farm” interviews were selected in a way to be representative of the various types of agricultural production systems in the Walloon Region (cereal cultivation, dairy and meat) and were organised in two clusters (a first cluster of 11 farmers with a high level of environmental practices and a second cluster of 23 farmers with conventional intensive production systems). This clustering was done so as to understand the effect of the contact with the network bridging organisation on the farmers' adhesion to the deep and medium measures in two different situations of environmental practices.

The quantitative part consisted of a telephone survey carried out from November 2013 to the beginning of February 2014 with 153 farmers. These farmers were selected to be representative of the various types of agricultural production systems in the Walloon Region (cereal cultivation, dairy and meat) and according to a purposive sampling amongst a list of farmers who have periodic contact with several of the collaborative network organisations discussed in the theory section above. Amongst the 153 farmers, one farmer was excluded because of bad interview conditions and two were excluded because of incomplete answers. 22 Farmers with a low level of contact and who participated in none of the 13 AEM's were also included as a control group. However, these were excluded from the statistical analysis through likewise deletion (cf. legend to Table 4). The various network bridging organisations, along with a set of other organisations that were selected as control variables, are listed in Table 3 for the specific case of the Walloon Region. The following four types of knowledge provision were considered to select the organisations in this table:

• Knowledge co-production and social learning with a public good orientation (the various organisations fulfilling some of the roles of network bridging organisations, such as multi-stakeholder collaborations by nature reserve organisations, Local Action Groups under the Leader programme of the EU or research partnerships) (Section 1. in Table 3)
• Transmission and dissemination of technical administrative knowledge for market cooperation (for example exchange of technical knowledge in cooperatives) (Section 2. in Table 3)
• Knowledge co-production and social learning with a private or club good orientation (for example professional federations and unions) (Section 3. in Table 3)
• Transmission and dissemination of technical administrative knowledge with a public good orientation (for example knowledge transmission by the implementation advisors of the government (*conseillers agro-environnementaux*)) (variable AE advisors in Table 4 below and Annex 1).

## 5 Results and Discussion

The results of the 34 qualitative field interviews, based on the common structured questionnaire, point to a positive role of network bridging organisations in the commitment to environmental practices. These results are reported in the electronic supplementary material to this article (cf. results and discussion of the structured field interviews).

To analyse the results of the quantitative survey the two following sub-models were developed:

• A first sub-model: to test for the impact of network bridging organisations on the adoption of deep and/or medium agri-environmental measures.
• A second sub-model: to test for the impact of network bridging organisations on change in practices by the farmer for implementing the MAE.

The results of the quantitative survey (cf. Table 4) are consistent with the findings of the qualitative survey. In particular, the statistical survey shows a significant correlation between participation in two major types of network bridging organisations (1.1. and 1.3. in Table 3) and both the adhesion to the scheme and the change in practices by the farmer when implementing the AEM measure. The collective actors, listed in Table 3, that had no specific environmental orientation or were oriented to advocacy instead of collaborative implementation actions, or to market cooperation only, did not show any significant correlation with the two outcome variables (i.e.: all the categories in Table 3, except for 1.1. and 1.3.).

### 5.1 Adhesion to Deep/Medium Environmental Measures

The results of the ADHESION M–D sub-model (first column of the outcome variables, Table 4) show that amongst the network organisations that were tested the fact that farmers had regular contacts with environmental network organisations (var. ENV. NETWORK ORG.: regional nature parks, river basin associations, stakeholder commissions of nature restoration projects and other government/non-profit environmental management networks) is significantly correlated with the uptake of deep and medium environmental measures. In contrast to the official governmental agri-environmental councillors or general technical advisors (cf. AE Advisors below), who provide the information needed to get into the schemes and understand the technical requirements, these organisations accomplish social networking and social learning related to environmental issues in general and are not part of the implementation process of the AEM scheme.

This result is consistent with the stylised facts on the implementation of the agri-environmental schemes reported in the literature, which highlight the positive role of landscape-related network organisations in an efficient participation in the agri-environmental measures (OECD, 2013, Juntti and Potter, 2002, Morris, 2006, Van Herzele et al., 2011; Lockie and Rockloff, 2004, Grasby, 2004, Curtis and De Lacy, 1996, Mues et al., 1998, ABARE, 2003, Curtis, 2003). In line with the role of non-profit organisations in social learning discussed above, the high amount of social learning that is required for these measures is mostly provided by organisations that have a clear environmental focus and facilitate social learning with a broad diversity of interdependent actors. Therefore, as expected, other social learning organisations listed in Table 3 do not show the same correlations (cf. the CETA/Comices which does not have this diversity and the GAL which does not have this environmental focus).

Although this was not the object of this study, some hints on the reasons – which are independent from the AEM scheme – why the farmers have searched contacts with these environmental management organisations can be gathered from the qualitative information collected in the interviews on the nature of each of the organisations with which the farmers have a contact (information provided for 114 out of the 505 organisations listed by the 152 farmers in the interviews). This information shows that farmers often get into contact with environmental management organisations in the context of specific services or projects unrelated to the specific measures of the AEM scheme as such (general information sessions, implication of farmers in nature reserve management plans, etc.). The latter is consistent with the literature discussed in the theory section on the complementarity between the governmental and non-governmental service providers. As explained above, non-governmental providers are particularly contacted when the issues require operating in a more open-ended non-coercive collaborative context which is the case for many complex environmental issues.

Contact with environmental advisors of the government (var. AE ADVISORS) is also not significantly correlated with adhesion. One reason for this is that under the current scheme, the AE advisors only had an information communication role, with the exception of the MAE 8,9 and 10, where a contact with the governmental environmental advisors is compulsory (GIREA, 2011). Collaboration with environmental research (var. COLLAB ENVT RESEARCH) is not significant; this may be related to the fact that this first sub-model only analyses the correlation with adhesion to the scheme and not with change in practices, which is more knowledge-intensive.

Regarding the other core explanatory variables of the model, the fact of being situated in a region with good soil and climatic conditions (in the study of the Walloon Region, these are the soils that are mainly situated to the north of the River Meuse, except for the Condroz regions which also have quite good soil conditions) (the variable CONDPEDO) is negatively correlated to adhesion to deep/medium measures. This indicates that farmers in the regions with less good soil conditions adhere more to the scheme. This is consistent with the fact that the measures explicitly target the maintenance of landscape components with a high environmental value (hedges, high natural value grasslands etc.), which are scarce in intensive agricultural grasslands (Aviron et al., 2005, Dahms et al., 2010, Uthes and Matzdorf, 2013). In addition, it has been shown that due to lower agricultural opportunity costs, peripheral, marginal and difficult-to-farm areas are particularly likely to be enrolled in the scheme (Evans and Morris, 1997). The sub-group of organic farmers that were part of the survey (18% of the sample, var. ORGANIC) shows a significant positive correlation to adhesion to deep/medium, which is as expected, as they have less effort to make in changing their practices for implementing one of the 9 deep/medium measures that were part of this survey. The same trend was also observed in Bonnieux and Dupraz, 2007, where a highly significant positive correlation was reported between the participation of farmers in a “payment by results” scheme and their experience with other agri-environmental schemes including organic farming. Finally, in relation to the basic economic variables of the model, farmers who experience high transaction costs for taking part in the scheme (TRANSACTION COSTS) show a negative correlation. Several studies point in the same direction, reporting that farmers tend to respond more positively to scheme prescriptions that are flexible (Gorton et al., 2008, Mazorra, 2001, Hodge, 2001, Mettepenningen et al., 2013). However, scheme flexibility does not come without its risks. Farmers could be tempted to choose options that involve relatively little change and incur limited costs, most likely bringing about few environmental gains (Hodge and Reader, 2010).

The variable NO CONTACT SALES REP is positive and significantly correlated with adhesion to deep/medium. This is consistent with the fact that famers who develop intensive farming practices have regular contacts with sales representatives. As shown elsewhere, these intensive farmers have higher opportunity costs in moving to environmentally demanding practices or adopting deep/medium measures (Crabtree et al., 1998, Wilson and Hart, 2000, Hartmann et al., 2006).

Regarding the control variables of the model, some of the variables related to the farmers' motivations to decide to step into the scheme are significant. The alignment with the farmers' CONCEPTION OF AGRICULTURAL PRACTICES as an important factor in the decision is negatively correlated with the adhesion to deep/medium measures. This is consistent with the fact that these measures by definition go further beyond his existing practices, as highlighted above in the description of these measures. The “lack of appropriate design to realise environmental objectives” as a motivation for not adhering to some measures (var. ENV OBJECTIVES REFUSAL) is correlated with a higher adhesion to the deep/medium measures. The latter might indicate that an unconvincing design of the scheme by the government plays a role in the refusal to adopt some of these measures, especially for the farmers who have a higher overall involvement with the scheme.

The remaining control variables are not significant. One important variable that is not significant is the degree of concern expressed over biodiversity by the farmer on a Eurobarometer scale (var. HIGH BIODIV CONCERN: eurobarometer, 2011, question QB3; and the environmental concern scale (ECS) adapted from Weigel and Weigel, 1978). In accordance with the results of Howley et al., 2014, there is no significant correlation between the expressed concern and adhesion to the scheme. This result is surprising considering that a high level of environmental awareness or environmental values amongst farmers is critical for biodiversity protection (Poláková et al., 2011, Potter et al., 1991, Macdonald and Johnson, 2000). Even though this variable only partially represents the framing of biodiversity issues by the farmers, the result probably suggests that some farmers who consider that biodiversity decrease is not amongst the most important environmental problem are nevertheless willing to step into service contracts. Moreover, as specified by Willock et al., 1999, attitudes and behaviour are not always linked. In particular, adhesion of the farmers is also related to a diversity of socially shared and publicly debated values and understandings, whose legitimacy might be increased through the contact with intermediary organisations, and not only to the individual farmer's attitudes (cf. for similar evidence along these lines, Kenter et al., 2014).

### 5.2 Change in Practice for Medium/Deep Measures

The second model (CHANGE IN PRACTICES M–D) compares the change in practices related to the AEM scheme by farmers who adopted at least one deep or medium measure as compared to absence of change in practice when adopting the deep or medium measures or the adoption of light measures only. The model considers a change in practice if the farmer states, for at least one medium or deep measure to which he adheres, that he changed certain practices on his farm for the implementation of that measure. If the MAE measures require the choice of an environmentally interesting land parcel, the model only considers “a change in practice” if the farmer explicitly confirms that he implemented the measure on an environmentally interesting land parcel.

The responses of farmers that have changed their practice when implementing medium or deep measures are significantly correlated with the variable “contacts with the environmental network organisations” (ENV. NETWORK ORG). The latter is in line with our hypothesis on social learning. However, in this second sub-model this group is also correlated with the variable “contacts in the context of environmental research organised by universities, governmental institutes or nature park managers” (var. COLLAB ENV. RESEARCH). These contacts are less frequent overall (16% out of the statistical sample, compared to 51% for ENV. NETWORK ORG, cf. table in the annex), but this result is consistent with the fact that for a substantial number of farmers the change in practices or the choice of the appropriate land parcel is demanding in terms of knowledge generation. In contrast to the previous model, there is also a significant correlation with the environmental advisors (var. AE Advisors) from the government, which seems to indicate the positive role of the environmental advisors in knowledge generation and social learning related to change in practices for the medium/deep environmental measures.

Most other core explanatory variables of the model show similar correlations as under the adhesion sub-model, except for one variable. The variable some fields in Natura 2000 is also positively correlated with change in practices. This can be related to the fact that a farmer who has some fields in Natura 2000 has fewer knowledge costs for further changing his agricultural practices, as he is already involved in biodiversity-related management for some of his parcels (in part related to the compulsory measures in Natura 2000 areas). This importance of the prior knowledge-related factor is consistent with other surveys (showing that previous participation in agri-environmental payments schemes leads to a higher rate of change of environmental practices (Dupraz et al., 2002)).

Finally most control variables show similar correlations as under the adhesion sub-model, except for two attitudinal variables that are now not significant. This is consistent with the literature highlighting the lack of attitudinal change generated by the “action-oriented” AEM schemes, as reported in Section 2.

## 6 Conclusion

A broad literature on natural resources policies has shown the potential of collaborative networks in fostering change in environmental practices in various areas. However, most of the empirical analysis pertaining to collaborative governance has focused on forms of formal co-management regimes between the state and the concerned actors on the one hand or on situations of co-governance where the stakeholders had only a consultative or advisory role to the state.

This paper explored the intermediary case of implementation of environmental policies through a combination of governmentally managed monetary incentive schemes and social learning processes organised by a broad set of network bridging organisations that foster cooperation and social learning amongst state and non-state collective actors around environmental management initiatives. To this end, both in-depth field research and a purposive sampling were organised amongst farmers that take part in such schemes and who are interacting with a broad range of networks of state and non-state collective actors. Two main lessons may be drawn from the analysis. First, in a situation where farmers get involved in social learning processes and knowledge co-production with other actors, there is a clear improvement both in adhesion to deep/medium measures (which have a higher level of environmental requirements) and in change in environmental practices directly related to the AEM scheme. This effect is observed both for farmers with high and low concern for biodiversity depletion. Second, these social learning processes appear to be especially strong when farmers have periodic contact with environmentally oriented network bridging organisations, while they are clearly not significant in case of periodic contacts with non-state collective actors that are oriented towards advocacy or market coordination.

## Figures and Tables

**Table:** 

Concept	Variable	= 1	Definition	q*
Dependent variables of the two sub-models				
Sub-model 1	Adhesion M–D	76	= 1 if the farmer adheres to at least one medium or one deep measure. = 0 adhesion to light measures only. (light, medium, deep: as defined in Table 1)	15,19,23,27,31,35,43,51,55
Sub-model 2	Change in practice M–D	38	= 1 if the farmer states, for at least one medium or deep measure that, for the implementation of that measure he changed certain practices in his farm AND has chosen an environmentally interesting land parcel when applicable. = 0 if these two statements never apply together for all medium and deep to which the farmer adheres or if he only adheres to light. The other statements that the farmer could select for the medium/deep measures to which he adheres are: he has been able to continue the same practices thanks to the AEM, he did not modify his practices, he develops other environmental practices unrelated to the AEM. For the choice of land parcel the other options are: choice of an unproductive land parcel and choice of a parcel where these practices already apply.	17,21,25,29,33,37,45,53,57,59

Network governance model for compensation payments				
Monitoring and technical advice from governt	AE Advisors	108	= 1 if the farmer has interactions with governmental agri-environmental councillors or general technical advisors (one of the 4 options on a scale from every day to a few days/year). = 0 if the farmer has no contact.	62
Network bridging organisations	Env. Network Org.	65	= 1 if the farmer has contact with an environmental management organisation (as defined in Table 3) at least two days a year. = 0 if the farmer has no contact with an environmental management organisation.*	62
	Collab Env. Research	21	= 1 the farmer has collaborated or collaborates in research related to the environment. = 0 if this is not the case.	63
On-farm ecological potential	Condpedo	83	= 1 if the farm is part of a region with excellent soil and climate conditions (Condroz, silty (loam) or sand/silty region). = 0 if the farm is part of another region with poorer conditions.	4
	Organic	27	= 1 if the farm is an organic farm. = 0 if this is not the case.	6
	Some fields in Natura 2000	63	= 1 if some fields are under Natura 2000 management scheme. = 0 if this is not the case.	6
Governt scheme of monetary payments	Compensation payt	50	= 1 if the fact that the level of compensation payment is important was the most important factor for the farmer to adhere to the scheme, for at least one of the measures to which he adhered. = 0 otherwise.	8,12,16,20,24,28,32,36,40,44,48,52,44,56
	Transaction costs	67	= 1 if one of the following transaction costs (lack of information, lack of technical specification, excessive control) has played a role in the decision for non-participation of at least 3 agri-environmental measures that can are applicable to the farm. = 0 otherwise.	10,14,18,22,26,30,34,38,42,46,50,54,58

Control variables				
Farmers' motivations related to the MAE measures	Conception Agricultural Pract	79	= 1 if “the fact that AEM corresponds to my way of thinking about agricultural practices plays an important role or the most important role in my decision to adhere” (this should be selected for at least one of the light measures to which he adheres AND at least for one of the medium to which he adheres AND at least for one of the deep to which he adheres. = 0 otherwise (when he selects “plays no role in my decision or only a very little role”).	8,12,16,20,24,28,32,36,40,44,48,52,44,56
	Env. objectives	77	= 1 if he adheres to light measures at least for one of the light measures he selects the option “the fact that the measures is appropriate to reach environmental objectives has an important role or the most important role in my decision to adhere” AND if he adheres to medium measures at least for one of the medium this is valid AND if he adheres to deep measures at lease for one of the deep this is valid. = 0 otherwise.	8,12,16,20,24,28,32,36,40,44,48,52,44,56
	Env. objectives refusal	8	= 1 if he refuses to adhere to light measures that are applicable to his farm, at least for one of the light measures he selects the option “the fact that the measures is appropriate to reach environmental objectives has an important role or the most important role in my decision not to adhere” AND if he refuses to adhere to medium measures at least for one of the medium this is valid AND if he refuses to adhere to deep measures at lease for one of the deep this is valid. = 0 otherwise.	10,14,18,22,26,30,34,38,42,46,50,54,58
General farmers' motivations	High biodiv concern	12	= 1 if loss of biodiversity is ranked as one of the 3 highest environmental concerns (Eurobarometer, 2011, QB3) and the farmer agrees that there is a high probability of future species extension (within a list of question based on the environmental concern scale, Weigel and Weigel, 1978). = 0 if this is not the case.	66, 67
Type of production system	Dairy	49	= 1 if dairy cows are ranked as the most important activity on the farm. = 0 if this is not the case.	5
	No contact sales rep.	16	= 1 the farmer does not have contact with a sales representative. = 0 if he has a contact (on a scale: from every day to some days/year).	62

q* = question number of the corresponding survey question in the annex

**Table 1:** AEM scheme in the Walloon Region of Belgium (with the exception of AEM11, specifically related to organic farming). Participation rate amongst a total number of 15,274 farmers, for year 2010 (table based on data from GIREA, 2011). The categorisation in light/medium/deep is not based on the difficulty of implementing the measure on a given farm, but on an assessment of the gap between the technical requirements of the measures and the general legal compulsory baseline (independently of the effort that a given farmer needs to make to implement the measures, which will be assessed in Section 5.2 below): Light/medium/deep measures: measures to implement a set of environmental farming practices (satisfying a set of environmental objectives) that go a little/moderately/far beyond the existing general legal compulsory requirements that specify the minimum level of good agri-environmental practices. Spatial targeting of the measures for specific ecological areas: an increase in payment is foreseen for application in ecologically valuable areas (1a, 1b, 1c, 2, 3a, 3b), specific ecological areas are required (3b) or approval of an environmental advisor is required (8, 9, 10).

			Main environmental objective	Compensation payment	Spatial targeting	Participation rate
Light AEM	AEM1a	Hedges	Strengthening the ecological network	50€/200 m	+	33%
	AEM3a⁎	Grass strips along crops	Strengthening the ecological network	900€/ha	+	13%
	AEM4	Winter catch crops	Water ecosystem protection	100€/ha	+	22%
	AEM1b	Isolated trees	Strengthening the ecological network	25€/10 el.	+	15%
Medium AEM	AEM1c	Ponds	Strengthening the ecological network	50€/el.	+	10%
	AEM2	Natural grasslands	Strengthening the ecological network	200€/ha	+	13%
	AEM3b	Strip of extensive grassland	Strengthening the ecological network/water ecosystem protection	900€/ha	++	7%
	AEM5	Extensive cereals	Water ecosystem protection	100€/ha	no	4%
	AEM6	Endangered breeds	Agricultural biodiversity	120€/cattle, 30€/sheep, 2000€/equine	/	< 4%
Deep AEM	AEM7	Low cattle density	Low input/low environmental impact production system	100€/ha	/	4%
	AEM8	Grasslands of high biological value	Natura 2000 habitats and other high nature value grassland	450€/ha	++	5%
	AEM9	Specific buffer strips	Strengthening the ecological network, water and soil protection, targeted species	1250€/ha	++	7%
	AEM10	AE action plan	Multi-environmental objectives	1000–3000€/farm	+++	< 4%

⁎ This measure has “medium” level technical specifications but was included in the light measures in this study, because for some of the farms in the research sample that received this payment not all the technical specifications were implemented in practice.

**Table 2:** Forms of co-governance between state and non-state collective actors for agri-environmental public good provision.

	Core features	Illustrative examples	Strengths/weaknesses
Co-management between state and community/user groups (citations in Berkes, 2009, Dedeurwaerdere, 2009)	Formalised arrangement for power sharing between the state and non-state collective actors, through devolution of power to communities (or user groups).	Indigenous communities' formal management of forests; joint forest management organisations	(+) Possibility to more fully exploit local expertise and knowledge, clear formal decision-making arrangements (−) Need for lengthy negotiations to set up these arrangements
Collaborative networks between state and non-state collective actors (cf. references and discussion in Section 2 above)	Formal and informal arrangements for horizontal cooperation in the provision of environmental public goods	Multi-stakeholder management of river basins; Agenda 21 initiatives in the context of the implementation of the Convention on Biological Diversity; Local Action Groups in the EU LEADER programme	(+) Flexible and adaptive, capacity to create high involvement informal/horizontal cooperation (−) Requires some negotiation to set up these arrangements, in particular to build trust with the centralised state
Advisory bodies to the regulatory/centralised state (Prager and Nagel, 2008)	Consultation by a regulatory/centralised state of non-state collective actors	Environmental forum in Germany (cf. text above); EU consultative processes in the context of the Common Agricultural Policy	(+) Easy to set up, improves the decision-making quality of the central state (−) Consultative: full decision-making power remains with the state

**Table 3:**
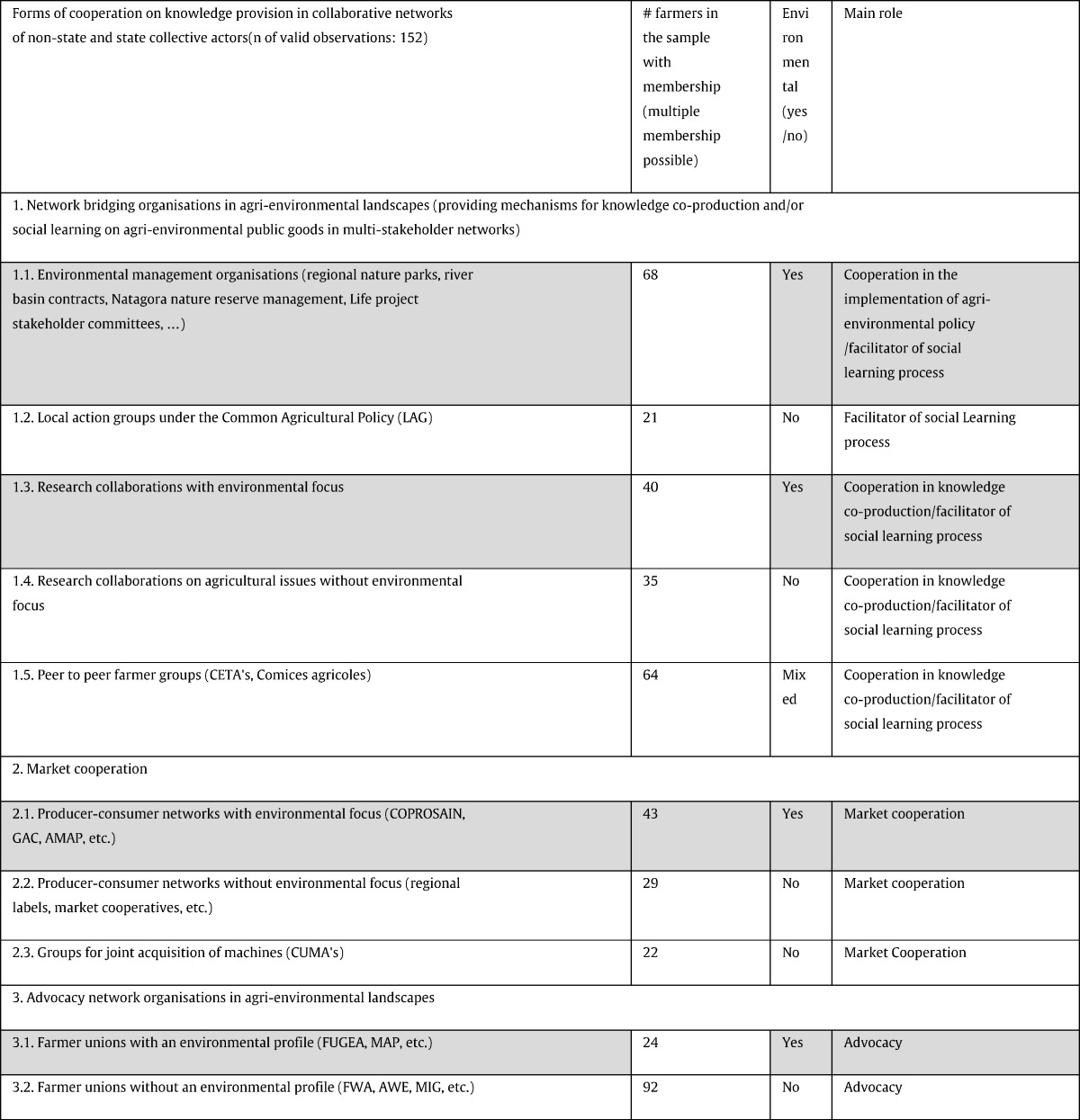
Overview of the main categories of organisations that provide knowledge and information to farmers, which were used in the questions on membership/participation by farmers in such organisations. Numbers are based on the quantitative survey (on a total of 152). One farmer can be a member of more than one organisation. Organisations with an explicit environmental focus are represented in grey.

Acronyms of organisations used in the table • GAC: Groupe d'achat collectif (collective acquisition of farm food baskets in direct producer consumer relationship) • AMAP: Association pour l'agriculture paysanne (same as GAC, but with a longer term contract with the farmers) • COPROSAIN: Coopérative de produits sains (a cooperative of sustainable farm products) • FUGEA: Fédération Unie de Groupements d'Eleveurs et d'Agriculteurs (Union of Farmers and Cattle Breeders) • MAP Mouvement d'action paysanne (Movement for peasant action) • FWA: Fédération wallonne de l'agriculture (Walloon Farmers' Union) • AWE: Association Wallonne de l'élevage (Walloon Association of Cattle Breeders) • MIG: Milcherzeuger Interessengemeinschaft (Union of Milk Producers) • CUMA: Coopérative d'utilisation de matériel agricole (Cooperative for the use of Agricultural Machinerie) • CETA: Centre d'études techniques agricoles (Centres for technical agricultural studies)

**Table 4:** Results of the biprobit models. Detailed discussion of the correlations and the variables follows in the text below; the technical description of the variables is presented in Annex 1. The questions of the structured questionnaire are provided in the electronic supplementary material. Results of the biprobit models. Detailed discussion of the correlations and the variables follows in the text below; the technical description of the variables is presented in Annex 1. The questions of the structured questionnaire are provided in the electronic supplementary material.

		Dependent variables of the two sub-models			
		Adhesion M–D	P > |z|	Change in practices M–D	P > |z|
		Adhesion to deep/medium measures as compared to adhesion to light measures only		For at least one medium or deep measure, the farmer both changed certain practices on his farm for the implementation of that measure and chose an environmentally interesting land parcel for that measure	

*Network governance model for compensation payments*					
Monitoring and technical advice from governt	AE advisors	**(+)**	0.389	(+)⁎⁎	0.020
Network bridging organisations	Env Network Org	**(+)**⁎⁎⁎	0.004	(+)⁎⁎⁎	0.005
	Collab Env Research	(+)	0.507	(+)⁎	0.096
On-farm ecological potential	Condpedo	(−)⁎⁎⁎	0.001	(−)⁎⁎⁎	0.002
	Organic	(+)⁎	0.063	(+)⁎⁎	0.029
	Some fields in Natura 2000	(+)	0.346	(+)⁎	0.074
Governt scheme of monetary payments	Compensation payt	(+)	0.665	(+)	0.513
	Transaction costs	(−)⁎⁎⁎	0.006	(−)⁎	0.093

*Control variables*					
Farmers' motivations related to the MAE measures	Conception agricultural pract	(−)⁎⁎	0.034	(−)	0.320
	Env objectives	(+)	0.362	(+)	0.862
	Env objectives refusal	(−)⁎	0.056	(+)	0.934
General farmers' motivations	High biodiv concern	(−)	0.647	(+)	0.898
Type of production system	Dairy	(−)	0.424	(+)	0.434
	No contact sales rep.	(+)⁎	0.071	(+)⁎⁎	0.015

Nb of observations = 128. Two of the 152 interviewees were excluded due to incomplete answers, 22 interviewees who adhered to no MAE were excluded due to likewise deletion in the statistical analysis, as the measurement of the variables Conception agricultural pract, Envt objectives, Env objective refusal and Change in practices M–D require to adhere at least to one MAE, cf table in Annex 1. Wald chi^2^(28) = 73.87; Prob > chi^2^ = 0.0000. Likelihood ratio test of rho21 = 0. Chi^2^(1) = 13.6573, Prob > chi2 = 0.0002.

Note that the table shows associations, not necessarily causal relationships. A bivariate probit (biprobit) system was estimated jointly for the two dependent variables (Adhesion M–D and Change in practices M–D). The Likelihood ratio test of rho21 confirms the choice of the bivariate probit framework. Conventional collinearity tests amongst the explanatory and control variables were conducted within Stata and showed no sign of collinearity amongst the variables (mean Variance Inflation Factor (VIF) = 1.24; SQRT VIF below 1.5 for all variables).

⁎ Significant at 90% level.

⁎⁎ Significant at 95% level.

⁎⁎⁎ Significant at 99% level.
