# Supplementary material for: The role of network bridging organisations in compensation payments for agri-environmental services under the EU Common Agricultural Policy
Source: Ecol Econ. 2015 Nov;119:24–38. doi: 10.1016/j.ecolecon.2015.07.025 (PMC5268349; doi:10.1016/j.ecolecon.2015.07.025)
Supplement: Supplementary file 1 — Supplementary material 1. [file mmc1.docx]

**Electronic supplementary material 1**

This electronic supplementary material presents the policy recommendations that result from the analysis in the paper.

***Policy recommendations***

The economic incentive policy of the agri-environmental payments has introduced a flexible scheme which can complement conventional direct governmental regulation. Even though many studies have focused on the environmental effects of this policy – which seems a difficult exercise given the multi-scale nature of the outcomes – few studies have analysed the impact of the way the scheme is governed on the commitment of farmers to change their agricultural practices.

The main result of the empirical surveys in the Walloon Region of Belgium discussed in this paper is that direct governmental control and monitoring of the scheme, without additional organisational means for fostering social learning and multi-actor cooperation, leads to ambiguous results in the implementation of the scheme. Currently the follow-up of farmers by the government is limited, even for farmers who implement the most demanding deep measures as they receive little advice from governmental AE advisors (normally 3 visits over the 5 years of contract, which is the legal requirement). However, farmers who have contacts with network bridging organisations (both for cooperation and social learning amongst the multiple stakeholders) clearly show a higher commitment to the scheme. Indeed, the bridging organisations (the research organisations and the environmental management organisations in the case of the Walloon survey) provide various services related to environmental management, but also opportunities for social learning amongst a diversity of concerned actors in a non-coercive environment, as illustrated in figure 4 of the article.

These results hint at an interesting policy outcome, which is the combination of agri-environmental payments with support for social learning and knowledge co-construction by network bridging organisations. The form that such policy integration could take is however a complex question for further research, in particular as governmental action should remain consistent with the open-ended and non-coercive character of social learning processes. However, given the role played already by these organisations in the Walloon Region, and given a number of other successful examples that have been reported in the literature (cf. section 1 above), this seems a promising way forward.

One option in this direction would be to examine, from a legal perspective, what are the options for synergies between the existing tools for social learning under the rural development component of the Common Agricultural Policy, such as the LEADER Programme (Local Action Groups), and the agri-environmental payment schemes. In one example in the Walloon Region, a Local Action Group also played a key role in the social learning process relating to the agri-environmental payment scheme (the case of CUESTAS asbl, Stassart, 2013), with very positive results. However, within the LEADER program, this possibility is not exploited in a systematic manner, which can also be seen in the lack of correlation in the quantitative survey between membership of Local Action Groups by farmers and their adhesion to deep environmental measures. From a legal perspective, a Local Action Group is only loosely defined: it has to develop activities that contribute to competitive agriculture, environmental protection or social and economic cohesion (Mestre et al., 2011). As a result, the legal requirements for the integration between these objectives are currently very weak.

In a similar way, support for network bridging organisations can be mobilised under the new EU regulation 1306/2013 concerning direct payments under the Common Agricultural Policy. This new regulation gives a broad interpretation of the farm advisory system, which has to support the implementation of the agri-environmental payments and the environmental conditionality under the regular direct payments to the farmers. At present, this regulation has been essentially used to support the implementation advisors (*conseillers agro-environnementaux*, cf. variable AE advisors), but in the future it could also accommodate support to the facilitation of knowledge co-production between the farmers, the non-state collective actors and the state actors through the network bridging organisations.

A second option would be to examine the legal grounds of policies in other countries where such a combined approach to social learning and economic incentives has been put into place. One prominent example already mentioned above is the highly successful agri-environmental policy in Quebec between 1997 and 2007, which was based on joint action on agri-environmental payments and support for farm-led competence groups (“club-conseils”) that organised a peer-to-peer social learning process for the implementation of these measures, in collaboration with competence centres situated at universities (Mathe and Rivaud, 2009). These and other examples show that the current fragmentation of the legal framework for environmental policy in the EU is not a fatal flaw, but can potentially evolve into a set of measures that address the social learning, scientific research and economic sustainability issues in a coordinated manner.

**References**

Mathe, J., and A. Rivaud. 2009. Les enjeux cognitifs du défi environnemental dans l’agriculture. XLVI ième colloque de l’Association de Science Régionale de Langue Française. Clermont-Ferrand (France), 6-8 juillet 2009.

Mestre, C., M. Blancquet et al. 2011. Politique agricole commune et politique commune de la pêche. Les éditions de l’Université de Bruxelles.

Stassart, P. 2013. Unlock the systemic locking ? From Conversion to Transition. Paper presented at the 1st Interdisciplinary Symposium on Sustainable Development, Namur, 31st of January 2013.
