# Supplementary material for: The role of network bridging organisations in compensation payments for agri-environmental services under the EU Common Agricultural Policy
Source: Ecol Econ. 2015 Nov;119:24–38. doi: 10.1016/j.ecolecon.2015.07.025 (PMC5268349; doi:10.1016/j.ecolecon.2015.07.025)
Supplement: Supplementary file 2 — Supplementary material 2. [file mmc2.docx]

**Electronic supplementary material 2**

This electronic supplementary material presents the results of the preliminary structured field interviews (addendum to first paragraph of section 5)

***Results and discussion of the preliminary structured field interviews***

The first group of farmers (11 interviewees) had both highly developed environmental practices (based on environmental indicators, cf. below) and intentions (farmers who explicitly stated that there was a need for a change to a more environmentally adequate agriculture system and who are actively informed on the applicable legislation). The farmers of this first group were organic farmers (7 out of 11) and low-input farmers with an equivalent high level of environmental practices (4 out of 11) (based on published reference indicators for rating environmental practices: low external inputs, soil conservation measures and increased crops/breed diversity (Peeters et al., 2004; Le Roux et al., 2012; IBIS, 2009; Guillaumin et al., 2007; Vilain, 2008). In contrast, the second group of farmers (23 interviewees) had a low level of environmental practices (according to the same indicators) or a low level of expressed intentions for the environmental transformation of their production system. All the farmers of this second group were conventional high-input farmers.

The following four core variables were addressed in the interviews: (1) the motivational role played by the amount of subsidies under the agri-environmental scheme, (2) the pro-environmental attitudes of the farmers, (3) the impact of their contacts with agri-environmental advisors from the government on their adhesion and environmental effort, (4) the impact of their contacts with non-profit environmental organisations or non-governmental individual advisors on their adhesion and environmental effort. At the stage of the qualitative study, these non-governmental organisations and advisors were diverse and included peer-to-peer farmers’ networks, independent environmental experts or lobbyists, coordinators of environmental research projects, natural reserves managers, and members of environmental organisations. It was only in the second stage of the research (the quantitative study) – and based on the outcomes of the first stage – that a more systematic approach of these organisations was elaborated as shown in table 3 above.

As can be expected, the farmers of the first group stated that the level of subsidies of the agri-environmental scheme was not the only reason why they decided to adopt deep and medium environmental measures. The objectives of the scheme, the way it is aligned or not with their vision of sustainable agriculture, were important motivational factors. In this group, 9 farmers adopted deep/medium measures and 2 organic farmers did not take part in the scheme. These latter 2 farmers, that did not adopt one the 10 MAE measures that are part of this survey (cf. table 1 above), in spite of their high level of environmental practices, disagreed with the compatibility of the scheme with organic farming systems. Nearly all farmers (10 out of 11) of this group had periodic contacts with network bridging organisations, such as research entities or environmental management organisations, and/or private environmental counsellors. In half of the cases, the farmers stated that these contacts had played a clear motivational role in their change in perception and understanding of environmental practices. In the other half of the cases, the contacts played a technical knowledge-supporting role.

Most farmers of the second group stated that the subsidies played a major role in their motivation to adopt certain measures or not to adopt other measures. Moreover, most of these farmers clearly indicated that they feared a productivity decrease through adhesion to agri-environmental measures. In this group, 14 farmers (out of 23) decided to adhere to medium or deep agri-environmental measures, but only 7 of these made an environmental effort, while the 7 others adhered without making an environmental effort. A substantial number of farmers that made an environmental effort of the sub-group of 14 had periodic contacts with network bridging organisations (such as research entities or environmental management organisations) and/or various ad hoc private environmental councillors (4 out of 7). In this sub-group, none of the farmers that made “no environmental effort” had a contact with such groups or individuals.

The results of the qualitative field interviews, based on the common structured questionnaire, point to a positive role of network bridging organisations in commitment to environmental practices. Farmers related this role explicitly to the social learning processes that changed their perceptions/understanding of environmental practices. However, this raises the question of whether the contact with the network bridging organisations resulted in an environmental effort or whether this effort would have occurred without these contacts. For the farmers of the first group, it was quite clear from the interviews that these contacts raised or at least maintained their motivation to make environmental efforts in the context of agri-environmental policy. For example, one farmer (20 June 2013, personal interview at Lessines, Belgium) of this group explained his adhesion to the agri-environmental scheme as a consequence of his collaboration with the manager of a nature reserve before participating in the agri-environmental measures. Both the information on the measures and the recommendations of the reserve manager on possible changes in his practices were key motivational factors that had gradually sensitised this farmer to environmental practices. Similar statements were made by the other farmers who adopted deep/medium measures in this first group, which hints at a strong causal role of the contacts with the advisors and/or network bridging organisations. The latter is further corroborated by the fact that, in the second group, almost all the farmers who adopted deep measures with an environmental effort both had periodic contacts with the network bridging organisations and report that these contacts had a direct impact on their motivation to change their agricultural practices.

Obviously, in spite of this consistent finding on the role of the network bridging organisations, important caveats do apply. First, this exploratory analysis of the 34 field interviews only addressed the contrasts between two farmer groups. Second, even though the farmers who adopted deep/medium measures in each of these groups report an impact of the bridging organisations on their environmental effort, the sample is too small to differentiate between the various types of bridging organisations. To this end we completed the field work by making a systematic approach to these organisations and a statistical phone interview amongst 153 farmers as described in the methods section above. The results of this second empirical study are reported in the core of the research paper.

**References**

Guillaumin, A., J. P. Hopquin, P. Desvignes, and J.M. Vinatier. 2007. Observatoire Territorial des Pratiques Agricoles (OTPA) : des indicateurs pour caractériser la participation des exploitations agricoles d’un territoire au développement durable. Deuxième partie : guide des indicateurs. 145 p.

IBIS. 2009. Intégrer la biodiversité dans les systèmes d’exploitations agricoles (IBIS). Diagnostic d’exploitation 48 p. <http://www.centre.chambagri.fr/cd_ibis/xdocs/pdf/IBIS_Guide_utilisation.pdf>

Le Roux, X., R. Barbault, J. Baudry, F. Burel, I. Doussan, F. Garnier et al. 2012. Agriculture et Biodiversité. Valoriser les Synergies. Expertise scientifique collective, INRA, France.

Peeters, A., J. F. Maljean, K. Biala, and V. Brouckaert. 2004. Les indicateurs de biodiversité pour les prairies : un outil d'évaluation de la durabilité des systèmes d'élevage. Fourrages: 217-232.

Vilain, L. 2008. La méthode IDEA, guide d’utilisation. Indicateurs de durabilité des exploitations agricoles.
