# Supplementary material for: The role of network bridging organisations in compensation payments for agri-environmental services under the EU Common Agricultural Policy
Source: Ecol Econ. 2015 Nov;119:24–38. doi: 10.1016/j.ecolecon.2015.07.025 (PMC5268349; doi:10.1016/j.ecolecon.2015.07.025)
Supplement: Supplementary file 4 — Supplementary material 4. [file mmc4.pdf]

Description of the Farm

1. Farmer identification

Last name, First name

Municipality

Address

Telephone

2. Identification on the sampling list.

3. Name of the interviewer.

4. Agricultural Region

- ○ Loamy region
- ○ Sandy-Loamy region
- ○ Condroz
- ○ Ardenne
- ○ Famenne
- ○ Jurassic
- ○ Peatland region
- ○ Grasland region
- ○ Upper-Ardenne

5. Please list the most significant activities on your farm (in terms of time, work and surface area involved). Please list them in order of importance (1 for the most important, 2 for the next most important, etc.).

Field crops

Dairy cattle rearing

Beef cattle rearing

Sheep rearing for milk

Sheep rearing for meat

Pork rearing for meat

Poultry

Horticulture

6. Please give a brief description of your farm according to the following indicators.

Agricultural land (in hectares): cash crops, feed crops (forage crops, cereals, etc.), permanent pastureland

Herd (LU or head, breed)

AEMs entered into (area or duration for each)

Natura 2000 parcel (yes/no, area)

Product label (quality, organic, etc.)

AEM 1a: Hedges

7. Do you have at least 200 metres of hedges? <u>No: please skip to the following page!</u> Yes: please answer this question: are you signed up to AEM1a, Hedges?

- ◯ I am not signed up
- ◯ I am not signed up but I have taken steps to sign up in the past
- ◯ I am not signed up but I used to be
- ◯ I am signed up

If you are signed up, from which year? <u>If you are not signed up, please read the last question on this page.</u>

____

8. If you have adopted AEM1a (Hedges), did the following factors play a part in your decision to adopt?

	No part	Very little part	Significant part	Most significant part
The AEM is appropriate to achieving environmental objectives	◯	◯	◯	◯
The AEM subsidy is worth having	◯	◯	◯	◯
The AEM corresponds to my way of thinking about agricultural practices	◯	◯	◯	◯
The information provided is clear and sufficient for implementation	◯	◯	◯	◯
The AEM specifications are sufficiently flexible	◯	◯	◯	◯
Signing up to the AEM does not result in too many inspections on my farm	◯	◯	◯	◯

9. If you have adopted AEM1a (Hedges) Adopting the AEM has brought about changes on your farm

- ☐ I have changed certain of my practices in order to implement the AEM
- ☐ I have developed other environmental practices not related to the AEM
- ☐ The subsidy has enabled me to maintain certain practices already in place on my farm
- ☐ I have not changed my practices since adopting the AEM

10. If you have not adopted AEM1a (Hedges), did the following factors play a part in your decision not to adopt?

	No part	Very little part	Significant part	Most significant part
The AEM is not appropriate to achieving environmental objectives	◯	◯	◯	◯
The AEM subsidy is not worth having	◯	◯	◯	◯
The AEM is not in keeping with my approach to agricultural practices	◯	◯	◯	◯
The information provided is not clear and sufficient for implementation	◯	◯	◯	◯
The AEM specifications are not flexible enough	◯	◯	◯	◯
Signing up to the AEM results in too many inspections on my farm	◯	◯	◯	◯

AEM1b : Isolated trees

11. Do you have at least 10 isolated trees on your land plots? No: please skip to the following page!
Yes: please answer this question: are you signed up to AEM1b, Isolated trees?

- ◯ I am not signed up
- ◯ I am not signed up but I have taken steps to sign up in the past
- ◯ I am not signed up but I used to be
- ◯ I am signed up

If you are signed up, from which year? If you are not signed up, please read the last question on this page.

12. If you have adopted AEM1b (Isolated trees), did the following factors play a part in your decision to adopt?

	No part	Very little part	Significant part	Most significant part
The AEM is appropriate to achieving environmental objectives	◯	◯	◯	◯
The AEM subsidy is worth having	◯	◯	◯	◯
The AEM corresponds to my way of thinking about agricultural practices	◯	◯	◯	◯
The information provided is clear and sufficient for implementation	◯	◯	◯	◯
The AEM specifications are sufficiently flexible	◯	◯	◯	◯
Signing up to the AEM does not result in too many inspections on my farm	◯	◯	◯	◯

13. If you have adopted AEM1b (Isolated trees)
Adopting the AEM has brought about changes on your farm

- ☐ I have changed certain of my practices in order to implement the AEM
- ☐ I have developed other environmental practices not related to the AEM
- ☐ The subsidy has enabled me to maintain certain practices already in place on my farm
- ☐ I have not changed my practices since adopting the AEM

14. If you have not adopted AEM1b (Isolated trees), did the following factors play a part in your decision not to adopt?

	No part	Very little part	Significant part	Most significant part
The AEM is not appropriate to achieving environmental objectives	◯	◯	◯	◯
The AEM subsidy is not worth having	◯	◯	◯	◯
The AEM is not in keeping with my approach to agricultural practices	◯	◯	◯	◯
The information provided is not clear and sufficient for implementation	◯	◯	◯	◯
The AEM specifications are not flexible enough	◯	◯	◯	◯
Signing up to the AEM results in too many inspections on my farm	◯	◯	◯	◯

AEM1c : Ponds

15. Do you have at least one pond on your land plots? No: please skip to the following page!
Yes: please answer this question: are you signed up to AEM1c, Ponds?

- ◯ I am not signed up
- ◯ I am not signed up but I have taken steps to sign up in the past
- ◯ I am not signed up but I used to be
- ◯ I am signed up

If you are signed up, from which year? If you are not signed up, please read the last question on this page.

[ ]

16. If you have adopted AEM1c (Ponds), did the following factors play a part in your decision to adopt?

	No part	Very little part	Significant part	Most significant part
The AEM is appropriate to achieving environmental objectives	◯	◯	◯	◯
The AEM subsidy is worth having	◯	◯	◯	◯
The AEM corresponds to my way of thinking about agricultural practices	◯	◯	◯	◯
The information provided is clear and sufficient for implementation	◯	◯	◯	◯
The AEM specifications are sufficiently flexible	◯	◯	◯	◯
Signing up to the AEM does not result in too many inspections on my farm	◯	◯	◯	◯

17. If you have adopted AEM1c (Ponds)
Adopting the AEM has brought about changes on your farm

- ☐ I have changed certain of my practices in order to implement the AEM
- ☐ I have developed other environmental practices not related to the AEM
- ☐ The subsidy has enabled me to maintain certain practices already in place on my farm
- ☐ I have not changed my practices since adopting the AEM

18. If you have not adopted AEM1c (Ponds), did the following factors play a part in your decision not to adopt?

	No part	Very little part	Significant part	Most significant part
The AEM is not appropriate to achieving environmental objectives	◯	◯	◯	◯
The AEM subsidy is not worth having	◯	◯	◯	◯
The AEM is not in keeping with my approach to agricultural practices	◯	◯	◯	◯
The information provided is not clear and sufficient for implementation	◯	◯	◯	◯
The AEM specifications are not flexible enough	◯	◯	◯	◯
Signing up to the AEM results in too many inspections on my farm	◯	◯	◯	◯

AEM2: Natural grasslands

19. Do you have permanent grasslands? No: please skip to the following page!
Yes: please answer this question: are you signed up to AEM2, Natural grasslands?

- ◯ I am not signed up
- ◯ I am not signed up but I have taken steps to sign up in the past
- ◯ I am not signed up but I used to be
- ◯ I am signed up

If you are signed up, from which year? If you are not signed up, please read the last question on this page.

20. If you have adopted AEM2 (Natural grasslands), did the following factors play a part in your decision to adopt?

	No part	Very little part	Significant part	Most significant part
The AEM is appropriate to achieving environmental objectives	◯	◯	◯	◯
The AEM subsidy is worth having	◯	◯	◯	◯
The AEM corresponds to my way of thinking about agricultural practices	◯	◯	◯	◯
The information provided is clear and sufficient for implementation	◯	◯	◯	◯
The AEM specifications are sufficiently flexible	◯	◯	◯	◯
Signing up to the AEM does not result in too many inspections on my farm	◯	◯	◯	◯

21. If you have adopted AEM2 (Natural grasslands)
Adopting the AEM has brought about changes on your farm

- ☐ I have changed certain of my practices in order to implement the AEM
- ☐ I have developed other environmental practices not related to the AEM
- ☐ The subsidy has enabled me to maintain certain practices already in place on my farm
- ☐ I have not changed my practices since adopting the AEM

22. If you have not adopted AEM2 (Natural grasslands), did the following factors play a part in your decision not to adopt?

	No part	Very little part	Significant part	Most significant part
The AEM is not appropriate to achieving environmental objectives	◯	◯	◯	◯
The AEM subsidy is not worth having	◯	◯	◯	◯
The AEM is not in keeping with my approach to agricultural practices	◯	◯	◯	◯
The information provided is not clear and sufficient for implementation	◯	◯	◯	◯
The AEM specifications are not flexible enough	◯	◯	◯	◯
Signing up to the AEM results in too many inspections on my farm	◯	◯	◯	◯

AEM3b: Grass strips in grasslands

23. Do you have permanent grasslands? <u>No: please skip to the following page!</u> Yes: please answer this question: are you signed up to AEM3b, Grass strips in grasslands?

- ◯ I am not signed up
- ◯ I am not signed up but I have taken steps to sign up in the past
- ◯ I am not signed up but I used to be
- ◯ I am signed up

If you are signed up, from which year? <u>If you are not signed up, please read the last question on this page.</u>

[ ]

24. If you have adopted AEM3b (Grass strips in grasslands), did the following factors play a part in your decision to adopt?

	No part	Very little part	Significant part	Most significant part
The AEM is appropriate to achieving environmental objectives	◯	◯	◯	◯
The AEM subsidy is worth having	◯	◯	◯	◯
The AEM corresponds to my way of thinking about agricultural practices	◯	◯	◯	◯
The information provided is clear and sufficient for implementation	◯	◯	◯	◯
The AEM specifications are sufficiently flexible	◯	◯	◯	◯
Signing up to the AEM does not result in too many inspections on my farm	◯	◯	◯	◯

25. If you have adopted AEM3b (Grass strips in grasslands) Adopting the AEM has brought about changes on your farm

- [ ] I have changed certain of my practices in order to implement the AEM
- [ ] I have developed other environmental practices not related to the AEM
- [ ] The subsidy has enabled me to maintain certain practices already in place on my farm
- [ ] I have not changed my practices since adopting the AEM

26. If you have not adopted AEM3b (Grass strips in grasslands), did the following factors play a part in your decision not to adopt?

	No part	Very little part	Significant part	Most significant part
The AEM is not appropriate to achieving environmental objectives	◯	◯	◯	◯
The AEM subsidy is not worth having	◯	◯	◯	◯
The AEM is not in keeping with my approach to agricultural practices	◯	◯	◯	◯
The information provided is not clear and sufficient for implementation	◯	◯	◯	◯
The AEM specifications are not flexible enough	◯	◯	◯	◯
Signing up to the AEM results in too many inspections on my farm	◯	◯	◯	◯

AEM6: Rare local breeds

27. Do you have permanent grasslands? No: please skip to the following page!
Yes: please answer this question: are you signed up to AEM6, Rare local breeds?

- ◯ I am not signed up
- ◯ I am not signed up but I have taken steps to sign up in the past
- ◯ I am not signed up but I used to be
- ◯ I am signed up

If you are signed up, from which year? If you are not signed up, please read the last question on this page.

28. If you have adopted AEM6 (Rare local breeds), did the following factors play a part in your decision to adopt?

	No part	Very little part	Significant part	Most significant part
The AEM is appropriate to achieving environmental objectives	◯	◯	◯	◯
The AEM subsidy is worth having	◯	◯	◯	◯
The AEM corresponds to my way of thinking about agricultural practices	◯	◯	◯	◯
The information provided is clear and sufficient for implementation	◯	◯	◯	◯
The AEM specifications are sufficiently flexible	◯	◯	◯	◯
Signing up to the AEM does not result in too many inspections on my farm	◯	◯	◯	◯

29. If you have adopted AEM6 (Rare local breeds)
Adopting the AEM has brought about changes on your farm

- ☐ I have changed certain of my practices in order to implement the AEM
- ☐ I have developed other environmental practices not related to the AEM
- ☐ The subsidy has enabled me to maintain certain practices already in place on my farm
- ☐ I have not changed my practices since adopting the AEM

30. If you have not adopted AEM6 (Rare local breeds), did the following factors play a part in your decision not to adopt?

	No part	Very little part	Significant part	Most significant part
The AEM is not appropriate to achieving environmental objectives	◯	◯	◯	◯
The AEM subsidy is not worth having	◯	◯	◯	◯
The AEM is not in keeping with my approach to agricultural practices	◯	◯	◯	◯
The information provided is not clear and sufficient for implementation	◯	◯	◯	◯
The AEM specifications are not flexible enough	◯	◯	◯	◯
Signing up to the AEM results in too many inspections on my farm	◯	◯	◯	◯

AEM7: Low cattle density

31. Do you have permanent grasslands? No: please skip to the following page!
Yes: please answer this question: are you signed up to AEM7, Low cattle density?

- ◯ I am not signed up
- ◯ I am not signed up but I have taken steps to sign up in the past
- ◯ I am not signed up but I used to be
- ◯ I am signed up

If you are signed up, from which year? If you are not signed up, please read the last question on this page.

32. If you have adopted AEM7 (Low cattle density), did the following factors play a part in your decision to adopt?

	No part	Very little part	Significant part	Most significant part
The AEM is appropriate to achieving environmental objectives	◯	◯	◯	◯
The AEM subsidy is worth having	◯	◯	◯	◯
The AEM corresponds to my way of thinking about agricultural practices	◯	◯	◯	◯
The information provided is clear and sufficient for implementation	◯	◯	◯	◯
The AEM specifications are sufficiently flexible	◯	◯	◯	◯
Signing up to the AEM does not result in too many inspections on my farm	◯	◯	◯	◯

33. If you have adopted AEM7 (Low cattle density)
Adopting the AEM has brought about changes on your farm

- ☐ I have changed certain of my practices in order to implement the AEM
- ☐ I have developed other environmental practices not related to the AEM
- ☐ The subsidy has enabled me to maintain certain practices already in place on my farm
- ☐ I have not changed my practices since adopting the AEM

34. If you have not adopted AEM7 (Low cattle density), did the following factors play a part in your decision not to adopt?

	No part	Very little part	Significant part	Most significant part
The AEM is not appropriate to achieving environmental objectives	◯	◯	◯	◯
The AEM subsidy is not worth having	◯	◯	◯	◯
The AEM is not in keeping with my approach to agricultural practices	◯	◯	◯	◯
The information provided is not clear and sufficient for implementation	◯	◯	◯	◯
The AEM specifications are not flexible enough	◯	◯	◯	◯
Signing up to the AEM results in too many inspections on my farm	◯	◯	◯	◯

AEM8: Grasslands of high biological value

35. Do you have permanent grasslands? No: please skip to the following page! Yes: please answer this question: are you signed up to AEM8, Grasslands of high biological value ?

- ◯ I am not signed up
- ◯ I am not signed up but I have taken steps to sign up in the past
- ◯ I am not signed up but I used to be
- ◯ I am signed up

If you are signed up, from which year? If you are not signed up, please read the last question on this page.

[ ]

36. If you have adopted AEM8 (Grasslands of high biological value), did the following factors play a part in your decision to adopt?

	No part	Very little part	Significant part	Most significant part
The AEM is appropriate to achieving environmental objectives	◯	◯	◯	◯
The AEM subsidy is worth having	◯	◯	◯	◯
The AEM corresponds to my way of thinking about agricultural practices	◯	◯	◯	◯
The information provided is clear and sufficient for implementation	◯	◯	◯	◯
The AEM specifications are sufficiently flexible	◯	◯	◯	◯
Signing up to the AEM does not result in too many inspections on my farm	◯	◯	◯	◯

37. If you have adopted AEM8 (Grasslands of high biological value) Adopting the AEM has brought about changes on your farm

- ☐ I have changed certain of my practices in order to implement the AEM
- ☐ I have developed other environmental practices not related to the AEM
- ☐ The subsidy has enabled me to maintain certain practices already in place on my farm
- ☐ I have not changed my practices since adopting the AEM

38. If you have not adopted AEM8 (Grasslands of high biological value), did the following factors play a part in your decision not to adopt?

	No part	Very little part	Significant part	Most significant part
The AEM is not appropriate to achieving environmental objectives	◯	◯	◯	◯
The AEM subsidy is not worth having	◯	◯	◯	◯
The AEM is not in keeping with my approach to agricultural practices	◯	◯	◯	◯
The information provided is not clear and sufficient for implementation	◯	◯	◯	◯
The AEM specifications are not flexible enough	◯	◯	◯	◯
Signing up to the AEM results in too many inspections on my farm	◯	◯	◯	◯

AEM3a: Grass strips along crops

39. Do you have land plots with crops? <u>No: please skip to the following page!</u> Yes: please answer this question: are you signed up to AEM3a, Grass strips along crops ?

- ◯ I am not signed up
- ◯ I am not signed up but I have taken steps to sign up in the past
- ◯ I am not signed up but I used to be
- ◯ I am signed up

If you are signed up, from which year? <u>If you are not signed up, please read the last question on this page.</u>

40. If you have adopted AEM3a (Grass strips along crops), did the following factors play a part in your decision to adopt?

	No part	Very little part	Significant part	Most significant part
The AEM is appropriate to achieving environmental objectives	◯	◯	◯	◯
The AEM subsidy is worth having	◯	◯	◯	◯
The AEM corresponds to my way of thinking about agricultural practices	◯	◯	◯	◯
The information provided is clear and sufficient for implementation	◯	◯	◯	◯
The AEM specifications are sufficiently flexible	◯	◯	◯	◯
Signing up to the AEM does not result in too many inspections on my farm	◯	◯	◯	◯

41. If you have adopted AEM3a (Grass strips along crops) Adopting the AEM has brought about changes on your farm

- ☐ I have changed certain of my practices in order to implement the AEM
- ☐ I have developed other environmental practices not related to the AEM
- ☐ The subsidy has enabled me to maintain certain practices already in place on my farm
- ☐ I have not changed my practices since adopting the AEM

42. If you have not adopted AEM3a (Grass strips along crops), did the following factors play a part in your decision not to adopt?

	No part	Very little part	Significant part	Most significant part
The AEM is not appropriate to achieving environmental objectives	◯	◯	◯	◯
The AEM subsidy is not worth having	◯	◯	◯	◯
The AEM is not in keeping with my approach to agricultural practices	◯	◯	◯	◯
The information provided is not clear and sufficient for implementation	◯	◯	◯	◯
The AEM specifications are not flexible enough	◯	◯	◯	◯
Signing up to the AEM results in too many inspections on my farm	◯	◯	◯	◯

AEM9: Managed field strips

43. Do you have land plots with crops? No: please skip to the following page! Yes: please answer this question: are you signed up to AEM9, Managed field strips?

- ◯ I am not signed up
- ◯ I am not signed up but I have taken steps to sign up in the past
- ◯ I am not signed up but I used to be
- ◯ I am signed up

If you are signed up, from which year? If you are not signed up, please read the last question on this page.

44. If you have adopted AEM9 (Managed field strips), did the following factors play a part in your decision to adopt?

	No part	Very little part	Significant part	Most significant part
The AEM is appropriate to achieving environmental objectives	◯	◯	◯	◯
The AEM subsidy is worth having	◯	◯	◯	◯
The AEM corresponds to my way of thinking about agricultural practices	◯	◯	◯	◯
The information provided is clear and sufficient for implementation	◯	◯	◯	◯
The AEM specifications are sufficiently flexible	◯	◯	◯	◯
Signing up to the AEM does not result in too many inspections on my farm	◯	◯	◯	◯

45. If you have adopted AEM9 (Managed field strips) Adopting the AEM has brought about changes on your farm

- ☐ I have changed certain of my practices in order to implement the AEM
- ☐ I have developed other environmental practices not related to the AEM
- ☐ The subsidy has enabled me to maintain certain practices already in place on my farm
- ☐ I have not changed my practices since adopting the AEM

46. If you have not adopted AEM9 (Managed field strips), did the following factors play a part in your decision not to adopt?

	No part	Very little part	Significant part	Most significant part
The AEM is not appropriate to achieving environmental objectives	◯	◯	◯	◯
The AEM subsidy is not worth having	◯	◯	◯	◯
The AEM is not in keeping with my approach to agricultural practices	◯	◯	◯	◯
The information provided is not clear and sufficient for implementation	◯	◯	◯	◯
The AEM specifications are not flexible enough	◯	◯	◯	◯
Signing up to the AEM results in too many inspections on my farm	◯	◯	◯	◯

AEM4: Winter cover of the soil between crops

47. Do you cultivate sometimes spring crops? No: please skip to the following page! Yes: please answer this question: are you signed up to AEM4, Winter cover of the soil between crops ?

- ◯ I am not signed up
- ◯ I am not signed up but I have taken steps to sign up in the past
- ◯ I am not signed up but I used to be
- ◯ I am signed up

If you are signed up, from which year? If you are not signed up, please read the last question on this page.

[ ]

48. If you have adopted AEM4 (Winter cover of the soil between crops), did the following factors play a part in your decision to adopt?

	No part	Very little part	Significant part	Most significant part
The AEM is appropriate to achieving environmental objectives	◯	◯	◯	◯
The AEM subsidy is worth having	◯	◯	◯	◯
The AEM corresponds to my way of thinking about agricultural practices	◯	◯	◯	◯
The information provided is clear and sufficient for implementation	◯	◯	◯	◯
The AEM specifications are sufficiently flexible	◯	◯	◯	◯
Signing up to the AEM does not result in too many inspections on my farm	◯	◯	◯	◯

49. If you have adopted AEM4 (Winter cover of the soil between crops) Adopting the AEM has brought about changes on your farm

- ☐ I have changed certain of my practices in order to implement the AEM
- ☐ I have developed other environmental practices not related to the AEM
- ☐ The subsidy has enabled me to maintain certain practices already in place on my farm
- ☐ I have not changed my practices since adopting the AEM

50. If you have not adopted AEM4 (Winter cover of the soil between crops), did the following factors play a part in your decision not to adopt?

	No part	Very little part	Significant part	Most significant part
The AEM is not appropriate to achieving environmental objectives	◯	◯	◯	◯
The AEM subsidy is not worth having	◯	◯	◯	◯
The AEM is not in keeping with my approach to agricultural practices	◯	◯	◯	◯
The information provided is not clear and sufficient for implementation	◯	◯	◯	◯
The AEM specifications are not flexible enough	◯	◯	◯	◯
Signing up to the AEM results in too many inspections on my farm	◯	◯	◯	◯

AEM5: Extensive cereal crops

51. Do you cultivate sometimes cereals? No: please skip to the following page!
Yes: please answer this question: are you signed up to AEM5, Extensive cereal crops ?

- ◯ I am not signed up
- ◯ I am not signed up but I have taken steps to sign up in the past
- ◯ I am not signed up but I used to be
- ◯ I am signed up

If you are signed up, from which year? If you are not signed up, please read the last question on this page.

52. If you have adopted AEM5 (Extensive cereal crops), did the following factors play a part in your decision to adopt?

	No part	Very little part	Significant part	Most significant part
The AEM is appropriate to achieving environmental objectives	◯	◯	◯	◯
The AEM subsidy is worth having	◯	◯	◯	◯
The AEM corresponds to my way of thinking about agricultural practices	◯	◯	◯	◯
The information provided is clear and sufficient for implementation	◯	◯	◯	◯
The AEM specifications are sufficiently flexible	◯	◯	◯	◯
Signing up to the AEM does not result in too many inspections on my farm	◯	◯	◯	◯

53. If you have adopted AEM5 (Extensive cereal crops)
Adopting the AEM has brought about changes on your farm

- ☐ I have changed certain of my practices in order to implement the AEM
- ☐ I have developed other environmental practices not related to the AEM
- ☐ The subsidy has enabled me to maintain certain practices already in place on my farm
- ☐ I have not changed my practices since adopting the AEM

54. If you have not adopted AEM5 (Extensive cereal crops), did the following factors play a part in your decision not to adopt?

	No part	Very little part	Significant part	Most significant part
The AEM is not appropriate to achieving environmental objectives	◯	◯	◯	◯
The AEM subsidy is not worth having	◯	◯	◯	◯
The AEM is not in keeping with my approach to agricultural practices	◯	◯	◯	◯
The information provided is not clear and sufficient for implementation	◯	◯	◯	◯
The AEM specifications are not flexible enough	◯	◯	◯	◯
Signing up to the AEM results in too many inspections on my farm	◯	◯	◯	◯

AEM10: Agri-environmental action plan

55. Are you signed up to more than two AEMs? <u>No: please skip to the following page!</u> Yes: please answer this question: are you signed up to AEM10, Agri-environmental action plan?

- ◯ I am not signed up
- ◯ I am not signed up but I have taken steps to sign up in the past
- ◯ I am not signed up but I used to be
- ◯ I am signed up

If you are signed up, from which year? <u>If you are not signed up, please read the last question on this page.</u>

56. If you have adopted AEM10 (Agri-environmental action plan), did the following factors play a part in your decision to adopt?

	No part	Very little part	Significant part	Most significant part
The AEM is appropriate to achieving environmental objectives	◯	◯	◯	◯
The AEM subsidy is worth having	◯	◯	◯	◯
The AEM corresponds to my way of thinking about agricultural practices	◯	◯	◯	◯
The information provided is clear and sufficient for implementation	◯	◯	◯	◯
The AEM specifications are sufficiently flexible	◯	◯	◯	◯
Signing up to the AEM does not result in too many inspections on my farm	◯	◯	◯	◯

57. If you have adopted AEM10 (Agri-environmental action plan) Adopting the AEM has brought about changes on your farm

- ☐ I have changed certain of my practices in order to implement the AEM
- ☐ I have developed other environmental practices not related to the AEM
- ☐ The subsidy has enabled me to maintain certain practices already in place on my farm
- ☐ I have not changed my practices since adopting the AEM

58. If you have not adopted AEM10 (Agri-environmental action plan), did the following factors play a part in your decision not to adopt?

	No part	Very little part	Significant part	Most significant part
The AEM is not appropriate to achieving environmental objectives	◯	◯	◯	◯
The AEM subsidy is not worth having	◯	◯	◯	◯
The AEM is not in keeping with my approach to agricultural practices	◯	◯	◯	◯
The information provided is not clear and sufficient for implementation	◯	◯	◯	◯
The AEM specifications are not flexible enough	◯	◯	◯	◯
Signing up to the AEM results in too many inspections on my farm	◯	◯	◯	◯

AEM adoption - implementation

59. In the case of the AEMs you have adopted, by what criteria did you choose the parcels on which to implement them? ***Please tick a maximum of two boxes for each AEM.***

	Unprofitable parcel	Environmental interest of the parcel	Already existing practice corresponding to the specifications of the AEM
AEM1 Hedges, Isolated trees or Ponds	☐	☐	☐
AEM2 Natural grasslands	☐	☐	☐
AEM3 Grass strips	☐	☐	☐
AEM4 Winter cover of the soil between crops	☐	☐	☐
AEM5 Extensive cereal crops	☐	☐	☐
AEM6 Rare local breeds	☐	☐	☐
AEM7 Low cattle density	☐	☐	☐
AEM8 Grasslands of high biological value	☐	☐	☐
AEM9 Managed field strips	☐	☐	☐
AEM10 Agri-environmental action plan	☐	☐	☐

Associations and social networks

60. What groups are you a member of, and for how long?

	No group membership	Less than 5 years	5 to 10 years	Over 10 years
Local marketing network or label (sales cooperative, direct producer-consumer networks, farmers' groups, organic labels, grouped sales, etc.)				
Organisations developing environmental projects (Natagora, Natural Parks, LIFE projects, GAL local action group projects, etc.)				
Unions and interest groups (FWA, FJA, FUGEA, MIG, etc.)				
Collaboration with research into agricultural issues				
Collaboration with research on environmental issues				
- Famers' groups (CETA, COMICE, etc.)				

61. How closely are you involved in these groups (please note your greatest involvement)?

	Membership only	Participation in activities	Attendance at coordination or management meetings
Local marketing network or label (sales cooperative, direct producer-consumer networks, famers' groups, organic label, grouped sales, etc.)			
Organisations developing environmental projects (Natagora, Natural Parks, LIFE projects, GAL local action group projects, etc.)			
Unions and interest groups (FWA, FJA, FUGEA, MIG, etc.)			
Collaboration with research into agricultural issues			
Collaboration with research on environmental issues			
Famers' groups (CETA, COMICE, etc.)			

62. How frequently do you meet the following?

	Every day	A few hours a week	A few days a month	A few days a year	No contact
Member of a farmers' group (CETA, COMICE, etc.)					
Environmental group (Natagora, Patrimoine Naturel, etc.)					
Sales reps					
Research centre (private, university, etc.)					
Government official (technician, AEM advisor)					
Union and farming interest group staff (FWA, FUGEA, MIG, etc.)					
Other local farmers					

63. Are you member of one of the following associations? (please create several lines if member of more than one association for a category)

	Involvement yes or no	Name of the association	Membership only	Membership and active partcipation	Membership and management meetings	Since 1 to 5 years	Since 5 to 10 years	Since 10 to 20 years	More than 20 years	During 1 year	During 1 to 5 years	During 5 to 10 years	More than 10 years
Envt management organisations	☐	☐	☐	☐	☐	☐	☐	☐	☐	☐	☐	☐	☐
Commercial organisation non-envt	☐	☐	☐	☐	☐	☐	☐	☐	☐	☐	☐	☐	☐
Commercial organisation envt	☐	☐	☐	☐	☐	☐	☐	☐	☐	☐	☐	☐	☐
Envt Unions	☐	☐	☐	☐	☐	☐	☐	☐	☐	☐	☐	☐	☐
Non-envt Unions	☐	☐	☐	☐	☐	☐	☐	☐	☐	☐	☐	☐	☐
Local action group	☐	☐	☐	☐	☐	☐	☐	☐	☐	☐	☐	☐	☐
Agricultural research	☐	☐	☐	☐	☐	☐	☐	☐	☐	☐	☐	☐	☐
Envt research	☐	☐	☐	☐	☐	☐	☐	☐	☐	☐	☐	☐	☐
Farmer collective machine buying group (CUMA)	☐	☐	☐	☐	☐	☐	☐	☐	☐	☐	☐	☐	☐
Farmer's group (CETA, COMICE)	☐	☐	☐	☐	☐	☐	☐	☐	☐	☐	☐	☐	☐

64. Please tick the persons who best correspond to the following statements (several responses possible).

	The following have already helped me solve agricultural problems on my farm	The following have already helped me to improve my environmental practices	The following have influenced my decision to sign up to one or more AEMs
Member of a farmers' group (CETA, COMICE, etc.)	☐	☐	☐
Environmental group (Natagora, Patrimoine Naturel, etc.)	☐	☐	☐
Sales reps	☐	☐	☐
Research centre (private, university, etc.)	☐	☐	☐
Government official (technician, AEM advisor)	☐	☐	☐
Union and farming interest group staff (FWA, FUGEA, MIG, etc.)	☐	☐	☐
Other local farmers	☐	☐	☐

65. . What kind of contact or activities do you share with the following categories of people?

	General information on environmental issues (information sessions, etc.)	Follow-up and personalised management of environmental practices (advice on the farm, etc.)	Involvement in projects with citizen or consumer groups (local action groups, local marketing, etc.)	Informing consumers about agricultural produce
Member of a farmers' group (CETA, COMICE, etc.)	☐	☐	☐	☐
Environmental group (Natagora, Patrimoine Naturel, etc.)	☐	☐	☐	☐
Sales reps	☐	☐	☐	☐
Research centre (private, university, etc.)	☐	☐	☐	☐
Government official (technician, AEM advisor)	☐	☐	☐	☐
Union and farming interest group staff (FWA, FUGEA, MIG, etc.)	☐	☐	☐	☐
Other local farmers	☐	☐	☐	☐

Environmental sensitivity

66. From the following list, please select the environmental problems that worry you most. Please list them in order of importance from 1 (most worrying) to 7 (least worrying).

- [ ] 1. Man-made catastrophes (oil spills, industrial accidents)
- [ ] 2. Health impacts of chemicals used in everyday products
- [ ] 3. Depletion of natural resources (oil, water, etc.)
- [ ] 4. Loss of biodiversity (disappearance of certain species, loss of wildlife, etc.)
- [ ] 5. Global warming (rising sea levels, warmer and more unstable climate, etc.)
- [ ] 6. Erosion of farmland (mud slides, flooding, etc.)
- [ ] 7. Destruction of traditional landscapes (motorway building, industrialised landscapes, etc.)

67. Please give your view on the following statements by indicating how far you agree with them on a scale of 0 (strongly disagree) to 4 (strongly agree).

	0	1	2	3	4
1. I would be willing to make personal sacrifices in order to reduce pollution, even if the short-term results were minimal.	○	○	○	○	○
2. Over coming decades, thousands of plant and animal species are going to disappear forever.	○	○	○	○	○
3. The benefits of modern products outweigh the pollution caused by their production and use.	○	○	○	○	○
4. Claims that we have a major influence over climate change are exaggerated.	○	○	○	○	○
5. Although there is constant contamination of our lakes, rivers and air, natural purifying processes will restore normal levels.	○	○	○	○	○
6. The government should provide every citizen with a list of agencies and bodies to which they could report the damage caused by pollution.	○	○	○	○	○
7. Industry is doing its best to develop effective anti-pollution technologies.	○	○	○	○	○
8. Were I to be asked, I would contribute time or money or both to an organisation (such as Natagora, etc.) to promote the quality of the environment.	○	○	○	○	○

68. Comments

Thank you for taking part !
