# Supplementary material for: The role of network bridging organisations in compensation payments for agri-environmental services under the EU Common Agricultural Policy
Source: Ecol Econ. 2015 Nov;119:24–38. doi: 10.1016/j.ecolecon.2015.07.025 (PMC5268349; doi:10.1016/j.ecolecon.2015.07.025)
Supplement: Supplementary file 5 — Supplementary material 5. [file mmc5.pdf]

Bivariate probit regression   Number of obs   =   128

Wald chi2(28)   =   73.87

Log likelihood = -87.636495   Prob > chi2   =   0.0000

	Coef.	Std. Err.	z	P>|z|	[95% Conf.	Interval]
ADH_MD						
AE_Advisor	.3117674	.3621783	0.86	0.389	-.398089	1.021624
Condpedo	-1.116289	.3406772	-3.28	0.001	-1.784004	-.4485737
Organic	.8696968	.4675012	1.86	0.063	-.0465887	1.785982
No_Contact_Sales_Rep	1.215428	.6724291	1.81	0.071	-.102509	2.533365
Dairy	-.2697975	.3376587	-0.80	0.424	-.9315964	.3920014
Natura_2000	.2841573	.3017709	0.94	0.346	-.3073028	.8756173
Collab_Env_Research	.3053076	.4605065	0.66	0.507	-.5972685	1.207884
High_Biodiv_concern	-.2440156	.5330768	-0.46	0.647	-1.288827	.8007956
Env_Network_Org	.960898	.3312449	2.90	0.004	.31167	1.610126
Transaction_Costs	-.8337859	.3025137	-2.76	0.006	-1.426702	-.2408699
Compensation payt	.1428506	.3294945	0.43	0.665	-.5029469	.788648
Concept_Agric_practices	-.874421	.4128947	-2.12	0.034	-1.68368	-.0651623
Envt_objectives	.3670894	.4026611	0.91	0.362	-.4221118	1.156291
Envt_objectives_Refusal	-1.515477	.7943651	-1.91	0.056	-3.072404	.0414504
_cons	.6592438	.4682852	1.41	0.159	-.2585785	1.577066
Change_MD						
AE_Advisor	1.050012	.4509293	2.33	0.020	.1662066	1.933817
Condpedo	-.9608091	.3153831	-3.05	0.002	-1.578949	-.3426696
Organic	.8114373	.3726813	2.18	0.029	.0809955	1.541879
No_Contact_Sales_Rep	1.342535	.5511012	2.44	0.015	.2623968	2.422674
Dairy	.2790147	.3567431	0.78	0.434	-.420189	.9782183
Natura_2000	.5933974	.3326256	1.78	0.074	-.0585369	1.245332
Collab_Env_Research	.7139369	.4288975	1.66	0.096	-.1266867	1.55456
High_Biodiv_concern	.0746816	.5820922	0.13	0.898	-1.066198	1.215561
Env_Network_Org	1.018362	.3624765	2.81	0.005	.3079215	1.728803
Transaction_Costs	-.5711641	.3405315	-1.68	0.093	-1.238594	.0962654
Compensation payt	-.2152694	.3288723	-0.65	0.513	-.8598472	.4293085
Concept_Agric_practices	-.3779383	.3802678	-0.99	0.320	-1.123249	.3673728
Envt_objectives	.0653615	.3771941	0.17	0.862	-.6739254	.8046484
Envt_objectives_Refusal	.058726	.7097192	0.08	0.934	-1.332298	1.44975
_cons	-1.866149	.5916633	-3.15	0.002	-3.025787	-.7065097
/athrho	15.76305	1220.849	0.01	0.990	-2377.056	2408.583
rho	1	9.92e-11			-1	1

Likelihood-ratio test of rho=0: chi2(1) = 13.6573 Prob > chi2 = 0.0002

Collinearity Diagnostics

Variable	VIF	SQRT VIF	Tolerance	R-Squared
AE_Advisor	1.22	1.10	0.8194	0.1806
Condpedo	1.43	1.20	0.6972	0.3028
Organic	1.28	1.13	0.7825	0.2175
No_Contact_Sales_Rep	1.28	1.13	0.7787	0.2213
Dairy	1.14	1.07	0.8754	0.1246
Natura_2000	1.15	1.07	0.8724	0.1276
Collab_Env_Research	1.14	1.07	0.8810	0.1190
High_Biodiv_concern	1.05	1.03	0.9508	0.0492
Env_Network_Org	1.19	1.09	0.8368	0.1632
Transaction_Costs	1.09	1.04	0.9182	0.0818
Compensation payt	1.22	1.11	0.8171	0.1829
Concept_Agric_practices	1.60	1.26	0.6260	0.3740
Envt_objectives	1.50	1.22	0.6685	0.3315
Envt_objectives_Refusal	1.10	1.05	0.9105	0.0895

Mean VIF 1.24

	Eigenval	Index
1	6.5807	1.0000
2	1.2943	2.2548
3	1.0527	2.5003
4	0.9968	2.5694
5	0.8670	2.7550
6	0.7869	2.8918
7	0.7036	3.0582
8	0.6303	3.2311
9	0.5606	3.4261
10	0.4394	3.8699
11	0.3571	4.2927
12	0.2636	4.9967
13	0.2228	5.4353
14	0.1697	6.2272
15	0.0745	9.3986

```
Condition Number         9.3986

Eigenvalues & Cond Index computed from scaled raw sscp (w/ intercept)

Det(correlation matrix)    0.2182
```
